# Restructuring of Epibacterial Communities on *Fucus vesiculosus* forma *mytili* in Response to Elevated *p*CO_2_ and Increased Temperature Levels

**DOI:** 10.3389/fmicb.2016.00434

**Published:** 2016-03-31

**Authors:** Birte Mensch, Sven C. Neulinger, Angelika Graiff, Andreas Pansch, Sven Künzel, Martin A. Fischer, Ruth A. Schmitz

**Affiliations:** ^1^Department of Biology, Institute for General Microbiology, Christian-Albrechts University KielKiel, Germany; ^2^Department of Applied Ecology and Phycology, Institute of Biological Sciences, University of RostockRostock, Germany; ^3^Coastal Ecology, Alfred Wegener InstituteList on the island of Sylt, Germany; ^4^Department of Evolutionary Genetics, Max Planck Institute for Evolutionary BiologyPlön, Germany

**Keywords:** ocean acidification, *p*CO_2_, global warming, metaorganism, epibacteria, *Fucus vesiculosus* forma *mytili*, 16S rDNA, mesocosm

## Abstract

Marine multicellular organisms in composition with their associated microbiota—representing metaorganisms—are confronted with constantly changing environmental conditions. In 2110, the seawater temperature is predicted to be increased by ~5°C, and the atmospheric carbon dioxide partial pressure (*p*CO_2_) is expected to reach approximately 1000 ppm. In order to assess the response of marine metaorganisms to global changes, e.g., by effects on host-microbe interactions, we evaluated the response of epibacterial communities associated with *Fucus vesiculosus* forma *mytili* (*F. mytili*) to future climate conditions. During an 11-week lasting mesocosm experiment on the island of Sylt (Germany) in spring 2014, North Sea *F. mytili* individuals were exposed to elevated *p*CO_2_ (1000 ppm) and increased temperature levels (Δ+5°C). Both abiotic factors were tested for single and combined effects on the epibacterial community composition over time, with three replicates per treatment. The respective community structures of bacterial consortia associated to the surface of *F. mytili* were analyzed by Illumina MiSeq 16S rDNA amplicon sequencing after 0, 4, 8, and 11 weeks of treatment (in total 96 samples). The results demonstrated that the epibacterial community structure was strongly affected by temperature, but only weakly by elevated *p*CO_2_. No interaction effect of both factors was observed in the combined treatment. We identified several indicator operational taxonomic units (iOTUs) that were strongly influenced by the respective experimental factors. An OTU association network analysis revealed that relationships between OTUs were mainly governed by habitat. Overall, this study contributes to a better understanding of how epibacterial communities associated with *F. mytili* may adapt to future changes in seawater acidity and temperature, ultimately with potential consequences for host-microbe interactions.

## Introduction

The surfaces of marine macroalgae offer a diverse substrate for attachment and are often colonized by a large variety of bacteria acquired from nearby macroalgae and from the surrounding waters (Lachnit et al., 2011; Egan et al., 2013; Stratil et al., 2013, 2014). It is further known that macroalgae affect and shape the biofilm formation and composition on its surface by either attracting or defending substances (Weinberger, 2007; Lachnit et al., 2010). Both, macroalgal host and microbiota mutually benefit from each other by e.g., nutrient exchange (Hellio et al., 2000; Goecke et al., 2010; Lachnit et al., 2010; Nasrolahi et al., 2012), and microbial biofilms attached to the surface offer a protective layer against e.g., settlement of larvae (Nasrolahi et al., 2012; Egan et al., 2013). Recent studies have shown that bacteria attached to macroalgal surfaces play important roles in the healthy development and life of their host (Egan et al., 2013; Singh and Reddy, 2014; Wichard, 2015). Moreover, Dittami and colleagues hypothesized optimized host-microbe-interactions to be essential for adaption to environmental changes (Dittami et al., 2015). In general, multicellular organisms have been recently recognized as “metaorganisms” comprising the eukaryotic host and its synergistic interdependence with microorganisms associated with the host (Bosch and McFall-Ngai, 2011; McFall-Ngai et al., 2013). Besides, the composition of the microbiota fundamentally depends on many biotic and abiotic factors, as well as numerous conditions (e.g., algal species, age, season, environmental parameters) as reviewed by Martin et al. (2014). Recent examples reported restructuring of marine bacterial communities on macroalgae in response to changing seawater temperature (Stratil et al., 2013), and pH-dependent community shifts on free-living marine bacteria even in response to small changes in pH due to elevated atmospheric carbon dioxide partial pressure (*p*CO_2_) levels (Krause et al., 2012). Besides, several other factors shaping microbial communities might be affected by environmental changes and need to be considered: e.g., the complex bacteria-bacteria interactions (cooperation and competition) within epibacterial communities, predatory bacteria like *Bdellovibrio* and like organisms (BALOs), or organic matter provided by the algal (recently reviewed by Dang and Lovell, 2016).

The atmospheric carbon dioxide (CO_2_) concentration constantly increases mainly as a result of human activities and dissolves in the oceans thus causing an increase in seawater acidity, designated as “ocean acidification” (Raven et al., 2005). As a second consequence, atmospheric CO_2_ acts as a greenhouse gas resulting in global warming (Levitus et al., 2000). According to the fifth IPCC report (RCP8.5) in 2110, the *p*CO_2_ is expected to reach ~1000 ppm and the seawater temperature is predicted to be increased by ~5°C (Raven et al., 2005; IPCC, 2013, 2014). To date, the impact of elevated *p*CO_2_ and increased temperature levels as predicted for the future ocean on marine bacterial communities remains poorly understood, in particular within complex benthic communities. The Sylt outdoor benthic mesocosm facility offers an ideal opportunity to simulate future underwater climate scenarios and thus to study their impacts on complex benthic communities on a large-scale (Stewart et al., 2013; Wahl et al., 2015; Pansch et al., 2016). Thus *p*CO_2_ and temperature in a benthic mesocosm experiment was manipulated in accordance to the described future climate predictions to study their single and combined impacts on a typical Wadden Sea benthic community including the key organism *Fucus vesiculosus* forma *mytili* (*F. mytili*).

*F. mytili* is a marine brown macroalga of the class *Fucophyceae* (*Phaeophyceae*) and is discussed to be a hybrid of the world-wide distributed *F. vesiculosus* and *F. spiralis* species (Nienburg, 1932; Coyer et al., 2006). However, *F. mytili* differs in many aspects from these two species (Albrecht, 1998). *F. mytili* typically grows on Wadden Sea mussel beds as found near the island of Sylt, thus is a key macroalgae for coastal ecosystems (Schories et al., 1997). To date the bacterial communities attached to the surface of North Sea *F. mytili* remained undescribed, as well as their response to changing environmental conditions. Here, the epibacterial community composition of *F. mytili* was investigated by 16S rDNA amplicon sequencing during a comprehensive 11-week benthic mesocosm experiment in spring 2014, in which *F. mytili* individuals were exposed to elevated *p*CO_2_ (1000 ppm) and increased temperature (+5°C) levels. Both abiotic stressors were tested for single and combined effects on the bacterial communities attached to the surface of *F. mytili* at the start and after 4, 8, and 11 weeks of treatment. In addition, the composition of free-living bacteria in the surrounding seawater was analyzed during the experiment. Several indicator operational taxonomic units (Fortunato et al., 2013) were identified for the observed *p*CO_2_ and temperature effects. In addition, we performed an OTU association network analysis to specifically address potential effects of the treatments on bacteria-bacteria interactions and thus examine the complex interactions among bacteria and their environment in a more comprehensive way. We further investigated the impacts of increasing seawater acidity and temperature on the *F. mytili* host individuals by determining relative growth rates and two physiological features, the carbon-to-nitrogen (C:N) ratio and the mannitol content, an important photoassimilate and storage compound in brown algae (Yamaguchi et al., 1966).

## Materials and methods

### The sylt outdoor benthic mesocosms

For a detailed description of the tidal benthic mesocosm facility on the island of Sylt (located at the Wadden Sea Station of the Alfred-Wegener-Institute in List, Germany) see Pansch et al. (2016). In short, the benthic mesocosm facility was constructed to simulate near-natural North Sea underwater climate scenarios. The outdoor system consists of 12 independent experimental units (constructed of black HDPE = high-density polyethylene, Figure S1), each with a seawater capacity of 1800 L and covered with slanted, translucent lids. For seawater sampling, side ports are available at ~40 cm water depth. To mimic Wadden Sea conditions, low/high tide was simulated by moving the gratings up/down and changing direction of seawater flow every 6 h. In order to ensure sufficient nutrient concentrations ~1800 L seawater were added daily to each tank. The non-filtered seawater was provided by a pipeline with its inlet located 50 m offshore. Prior to distribution into the mesocosms, the seawater was transferred into storage tanks inside the institute to remove sediment particles. Seawater overflow was directed back into the sea.

### Estimation of *p*CO_2_, measurements of water parameters and nutrients

The actual *p*CO_2_ was calculated from weekly measurements of total alkalinity (TA), acidity (total pH; see below), salinity and temperature using the CO2sys EXCEL Macro spreadsheet developed by Pierrot et al. (2006) for describing the marine carbonate system. The Multi Parameter Measurement System continuously measured seawater temperature and pH in the tanks. The pH was measured on NBS scale at 25°C (NBS = National Bureau of Standards; Dickson, 1984). Total pH values were calculated from NBS to total pH scale using the following equation: pH(x) = 8.0939 + ((E_*s*_ − E_*x*_)/0.05916) with E_*s*_ = mV Dickson Tris buffer/1000, and E_*x*_ = mV sample at 25°C/1000. Additional samples to determine TA and to measure salinity were taken on a weekly basis (*n* = 3), as well as water samples for the measurement of inorganic nutrient concentrations of silicate (SiO${}_{4}^{4-}$), ammonium (NH${}_{4}^{+}$), phosphate (PO${}_{4}^{3-}$), total nitrogen oxide (NO_*x*_), and nitrite (NO${}_{2}^{-}$) by spectrophotometry. Respective nitrate (NO${}_{3}^{-}$) concentrations were calculated as NO_*x*_ − NO${}_{2}^{-}$. For a detailed description of sampling and measurement procedures see (Pansch et al., 2016). All data concerning the water parameters and nutrients are provided as Supplementary Material (Figures S2, S3, respectively).

### Experimental setup and treatments

In early April 2014, individual *F. mytili* thalli were collected in a Wadden Sea mussel bed (at 55°01′42.2″N 8°25′59.4″E). Several thalli (~15) were bundled with wire rope resulting in voluminous bundles with ~130 g wet weight on average. Eleven of these *F. mytili* bundles, hereinafter referred to as *F. mytili* individuals, were fixed on top of the 1.0 m^2^ grating inside of each tank. In addition, several organisms commonly found in the natural habitat of *F. mytili* (the blue mussel *Mytilus edulis*, the Pacific oyster *Crassostrea gigas*, the periwinkles *Littorina littorea* and *L. mariae*, and amphipods of the genus *Gammarus* spp.) were added consistently in defined biomass to the 12 tanks.

*F. mytili* individuals were incubated in the benthic mesocosms under four different conditions: (1) increased temperature (+5°C temperature at ambient *p*CO_2_), (2) elevated CO_2_ (1000 ppm *p*CO_2_ at ambient temperature), (3) increased temperature and elevated CO_2_ (+5°C temperature at 1000 ppm *p*CO_2_), and (4) ambient control (ambient temperature and ambient *p*CO_2_) with three tanks per treatment. As described in detail by Pansch et al. (2016), the seawater temperature in the tanks was monitored by internal sensors and automatically controlled by either heating rods or cooling units, to keep a delta of +5 °C in relation to the ambient control. The seawater *p*CO_2_ was manipulated by continuous injections of 1000 ppm pre-mixed CO_2_ gas (pure CO_2_ plus compressed air) directly into the water column. The lid ensured similar atmospheric and seawater *p*CO_2_levels. The temperature in the tanks was simulated based on two adjustable sinus curves (year and day), checked and adjusted based on field measurements at least once a week. The simulated temperature equals the measured seawater temperature in the tanks due to rapid temperature adjustment.

### Sampling of *F. mytili* biofilm and surrounding waters

Prior to sampling, the gratings carrying the *F. mytili* individuals were stopped shortly before low tide to keep the *F. mytili* individuals covered with seawater during the sampling procedure. Seawater and biofilm samples were taken at the beginning (t0) and after 4, 8, and 11 weeks of incubation (2014/04/09, 05/08, 06/05, and 06/25). Planktonic cells of the surrounding waters (1 L) were collected via vacuum filtration of the seawater through 0.2 μm Millipore Express PLUS polyethersulfone membrane filters (Millipore, Billerica, MA, USA) at max. −0.2 bar vacuum. Prior to biofilm sampling, the *F. mytili* thalli surfaces to be sampled were rinsed with 0.22 μm filtered seawater to remove loosely attached cells and particles. Subsequently, swabs were taken from the surfaces (~15 cm^2^ by visual estimation) using sterile cotton swabs. Swabs and filters were placed on ice during sampling and stored at −80°C until DNA extraction. Importantly, during the experiment biofilm samples were taken from the same *F. mytili* individuals (one *F. mytili* at a defined position on each grating) from the upper, younger parts of several thalli. To prevent resampling of the same region, sampled thalli were labeled with small cable straps.

### DNA extraction and PCR amplification of bacterial 16S rDNA for illumina MiSeq amplicon sequencing

Genomic DNA (gDNA) was isolated using the Isol-RNA Lysis Reagent (5 PRIME, Gaithersburg, MD, USA) according to the manual section “Isolation of genomic DNA” with small modifications: Only half the volume of each component was used and the speed of centrifugation steps was generally increased to 12,000 x g. Cotton tips and membrane filters were removed prior to chloroform addition. The hypervariable region V1-V2 of the bacterial 16S rDNA was amplified from gDNA (~100 ng μL^−1^) using the primer set 27-forward and 338-reverse (Youssef et al., 2010). Beside the target-specific region each primer sequence contained a linker sequence, an 8-base identifier index and the Illumina specific region P5 (forward primer) or P7 (reverse primer), respectively, as recently described by Kozich et al. (2013). The PCR reaction mixture and amplification conditions were performed as described by Löscher et al. (2015). The PCR products were checked for correct size (~350 bp amplicon length) and band intensity, then correct amplicons were purified from 1% agarose gels using the MinElute Gel Extraction Kit (Qiagen, Hilden, Germany). The purified amplicons were quantified using a NanoDrop 1000 spectrophotometer (Thermo Fisher Scientific, Waltham, MA, USA), pooled in equimolar ratio and sequenced according to the manufacturer's protocol on a MiSeq Instrument using the MiSeq reagent Kit V3 chemistry (Illumina, San Diego, CA, USA). Sequences were submitted to the NCBI Sequence Read Archive under accession number SRP069256.

### Bioinformatic processing

Sequence processing was performed using mothur version 1.34.4 (Schloss et al., 2009; Kozich et al., 2013). Raw reads were concatenated to 9,364,598 contiguous sequences (contigs) using the command *make.contig*. Contigs with ambiguous bases or homopolymers longer than 8 bases as well as contigs longer than 552 bases were removed using *screen.seqs*. The remaining 8,354,564 contigs were screened for redundant sequences using *unique.seqs* and clustered into 2,537,511 unique sequences. The sequences were consecutively aligned (with *align.seqs*) to a modified version of the SILVA database release 102 (Pruesse et al., 2007) containing only the hypervariable regions V1 and V2. Sequences not aligning in the expected region were removed from the dataset with *screen.seqs*. The alignment was condensed by removing gap-only columns with *filter.seqs*. The final alignment contained 8,288,816 sequences (2,511,577 unique) of lengths between 253 and 450 bases. Rare and closely related sequences were clustered using *unique.seqs* and *precluster.seqs*. The latter was used to include sequences with up to three positional differences compared to larger sequence clusters into the latter. Chimeric sequences were removed using the Uchime algorithm (Edgar et al., 2011) via the command *chimera.uchime*, followed by *remove.seqs*. This left 7,934,922 sequences (163,446 unique) in the dataset. Sequence classification was performed using the Wang Method (Wang et al., 2007) on a modified Greengenes database (containing only the hypervariable regions V1 and V2) with a bootstrap threshold of 80%. Sequences belonging to the kingdom archaea, to chloroplasts or mitochondria were removed from the dataset using *remove.lineage*. OTUs (operational taxonomic units) were formed using the average neighbor clustering method with *cluster.split*. Parallelization of this step was done taking the taxonomic classification on the order level into account. A sample-by-OTU table containing 55,378 OTUs at the 97% level was generated using *make.shared*. OTUs were classified taxonomically using the modified Greengenes database mentioned above and the command *classify.otu*.

### Statistics on bacterial 16S rDNA amplicon data

Statistical downstream analysis was performed with custom scripts in R v3.1.3 (R Core Team, 2015). OTUs of very low abundance only increase computation time without contributing useful information. They were thus removed from the dataset as follows: After transformation of counts in the sample-by-OTU table to relative abundances (based on the total number of reads per sample), OTUs were ordered by decreasing mean percentage across samples. The set of ordered OTUs for which the cumulative mean percentage amounted to 95% was retained in the filtered OTU table, resulting in a decrease in the number of OTUs from 55,378 to 4,157.

The extent of change in relative OTU abundance across samples explained by the experimental factors Temperature, *p*CO_2_, Time, and Sample Type (see Table S1) was explored by redundancy analysis (RDA) with Hellinger-transformed OTU counts (Stratil et al., 2013, 2014; Langfeldt et al., 2014) using function *rda* of R package *vegan* v2.4-0 (Oksanen et al., 2015).

Model selection started with a full RDA model containing all main effects and interactions of experimental factors, using the following model formula:

$$\begin{array}{l} \text{Transformed OTU counts}\sim (\text{Type}\cdot \text{Temp}\cdot {\text{CO}}_{2})\\ \;+\;(\text{Type}\cdot \text{Temp}\cdot {\text{CO}}_{2}):\text{Week} \end{array}$$

The Week main effect was omitted from the formula as temporal effects were nested within other levels or their combinations. (Inclusion of a Week main effect would be justified if measures for factor Week were completely independent rather than repeated). A permutation scheme for permutation-based significance tests was chosen with function *how* of R package *permute* v0.8-4 (Simpson, 2015) to reflect the repeated-measures design as well as the temporal nature of factor Week. Permutation of samples within a sample unit (a set of repeated measures taken for a particular combination of Type, Temperature and *p*CO_2_ at the different time points of Week; see Table S1) was set to “series,” with the same permutation used for each sample unit; clusters of samples belonging to different sample units were allowed to be permuted freely.

The full model was simplified by backward selection with function *ordistep*. The final RDA model exhibited significant interaction effects CO_2_:Week and Type:Temp:Week (see Results Section). It was thus necessary to evaluate (i) the Temperature effect within each level of Type and Week and (ii) the *p*CO_2_ treatment effect within each level of Week. For (i) the effect of the Temperature:Week interaction was evaluated within each level of factor Type in appropriate submodels of the final RDA model; upon significance, the Temperature effect was further evaluated within each level of factor Week (within the same level of factor Type). For (ii) the *p*CO_2_ effect was evaluated within each level of factor Week in appropriate submodels of the final RDA model. *p*-values for each stage of these hierarchical testing schemes were corrected for multiple testing by Benjamini–Hochberg correction (false discovery rate, FDR; Benjamini and Hochberg, 1995). For each significant submodel, OTUs were determined that were significantly correlated with any axis in the RDA submodel by function *envfit* with 10^5^ permutations, followed by Benjamini–Hochberg correction. In order to reduce the number of tests in this procedure, OTUs were pre-filtered according to their vector lengths calculated from corresponding RDA scores (scaling 1) by profile likelihood selection (Zhu and Ghodsi, 2006).

OTUs significant at an FDR of 5% were further subject to indicator analysis with function *multipatt* of the R package *indicspecies* v1.7.5 (De Cáceres and Legendre, 2009) with 10^5^ permutations. **Indicator OTUs (iOTUs)**—in analogy to indicator species *sensu* De Cáceres and Legendre (2009)—are OTUs that prevail in a certain sample group (here: either a level of *p*CO_2_ within a certain level of Week, or a level of Temperature within a certain sample Type and level of Week) while being found only irregularly and at low abundance in other sample groups.

3D visualizations of the final RDA model were produced in kinemage format (Richardson and Richardson, 1992) using the R package *R2Kinemage* developed by S.C.N., and displayed in KiNG v2.21 (Chen et al., 2009). For alpha diversity analysis, effective OTU richness (Shannon numbers equivalent, ^1^D; Jost, 2006, 2007) was calculated from the filtered OTU table. Multi-panel alpha diversity and iOTU plots were drawn with R package *lattice* v0.20-33 (Sarkar, 2008).

An **OTU association network** was inferred with the R package *SpiecEasi* v0.1 (Kurtz et al., 2015). OTUs selected for network analysis were required to be present in ≥60% of all samples to reduce the number of zeros in the data for a robust calculation, resulting in a set of 97 OTUs. Calculations were performed with the Meinshausen and Bühlmann neighborhood selection framework (MB method; Meinshausen and Bühlmann, 2006). Correlations between associated OTUs were determined from the centered log-ratio-transformed counts. The *igraph* package v1.0.1 (Csárdi and Nepusz, 2006) was employed for visualizing moderate to strong OTU associations (absolute correlation ≥0.6).

### Relative biomass growth rates of *F. mytili* macroalgal hosts

Biomass growth of all *F. mytili* individuals was measured prior to and after the experiment (*n* = 11 per treatment) as wet weight. Biomass change (described by the relative growth rate RGR) for wet mass was calculated according to Lüning et al. (1990) using the formula:

(1) $$RGR\;\left(\%\;d^{-1}\right)=100\;\frac{ln(m_{t})-ln(m_{0})}{t}$$

where *m*_0_ represents the initial wet mass (g) and *m*_*t*_ the wet mass (g) after *t* days (d).

### *F. mytili* algal tissue sampling and physiological analysis (C:N, mannitol)

In order to assess the physiological parameters of *F. mytili* individuals after growing for 11 weeks under the four different treatments the thallus material was cut, cleaned of epibiota and freeze-dried for further analyses (*n* = 3 per treatment; same individuals used for biofilm sampling). Prior to the experiment, thallus material without visible epiphytes of six initial *F. mytili* individuals was freeze-dried to document the initial physiological status of *F. mytili* in its native habitat. For analyzing carbon (C) and nitrogen (N) contents, freeze-dried algal material was ground to powder using mortar and pistil. Three subsamples of 2 mg from each vegetative apex were loaded and packed into tin cartridges (6 × 6 × 12 mm). These packages were combusted at 950°C and the absolute contents of C and N were automatically quantified in an elemental analyzer (Elementar Vario EL III, Germany) using acetanilide as standard according to Verardo et al. (1990). Mannitol was extracted from three powdered subsamples of 10–20 mg freeze-dried alga material, and quantified by HPLC as described by Karsten et al. (1991).

### Statistical analysis of *F. mytili* macroalgal data

In order to evaluate the interaction effect of temperature and *p*CO_2_ on all variables measured (relative biomass growth rates, C:N ratio, C, N and mannitol content) at the end of the experiment, two-way ANOVAs were used with temperature and *p*CO_2_ as fixed factors. When the analysis did not show significant interactions, a one-way ANOVA was carried out for each factor separately. When the one-way ANOVA revealed significant differences, a *post hoc* Tukey's honest significant difference test was applied. Prior to the use of ANOVAs, data were tested for normality with the Kolmogorov-Smirnov and for homogeneity with the Levene's test. Data were analyzed using SPSS Statistics 20 (IBM, Armonk, NY, USA).

## Results

### Bacterial community patterns depended on the sample type and varied in time

**Figure 1:**
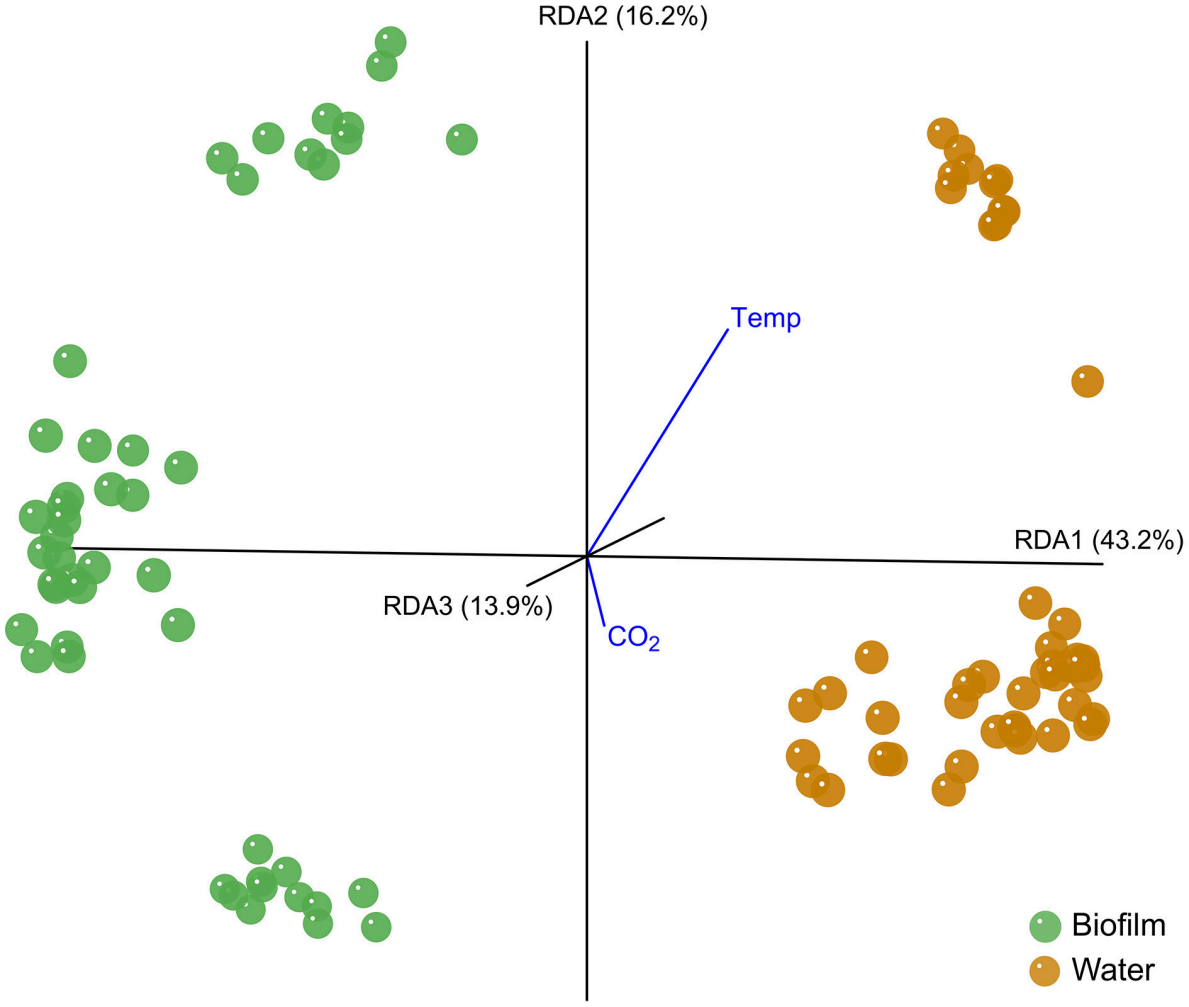
**Distance biplot of the type effect**. The type effect on *Fucus mytili* biofilm (green) and water (orange) samples is visualized with KiNG at RDA axes RDA1 43.2%, RDA2 16.2%, and RDA3 13.9% explained variance. Temperature and CO_2_ treatment effects are visualized as vectors pointing in negative direction of axis RDA3 (away from the observer). The analysis is based on MiSeq V1-V2 bacterial 16S rDNA amplicon sequence data on 97% sequence similarity.

The bacterial community pattern of *F. mytili* biofilm and water samples strongly varied from each other (Biofilm *F*_(11, 36)_ = 3.91, *p* = 0.003; Water *F*_(11, 36)_ = 7.06, *p* = 0.001; Table S2). A distance biplot visualizing the general type effect throughout the experiment is shown in Figure 1, with samples of *F. mytili* biofilm and water forming separated clusters. Variance partitioning showed that the type effect alone (i.e., with the influence of other variables removed) explained ~36% of the variance in the dataset.

**Figure 2:**
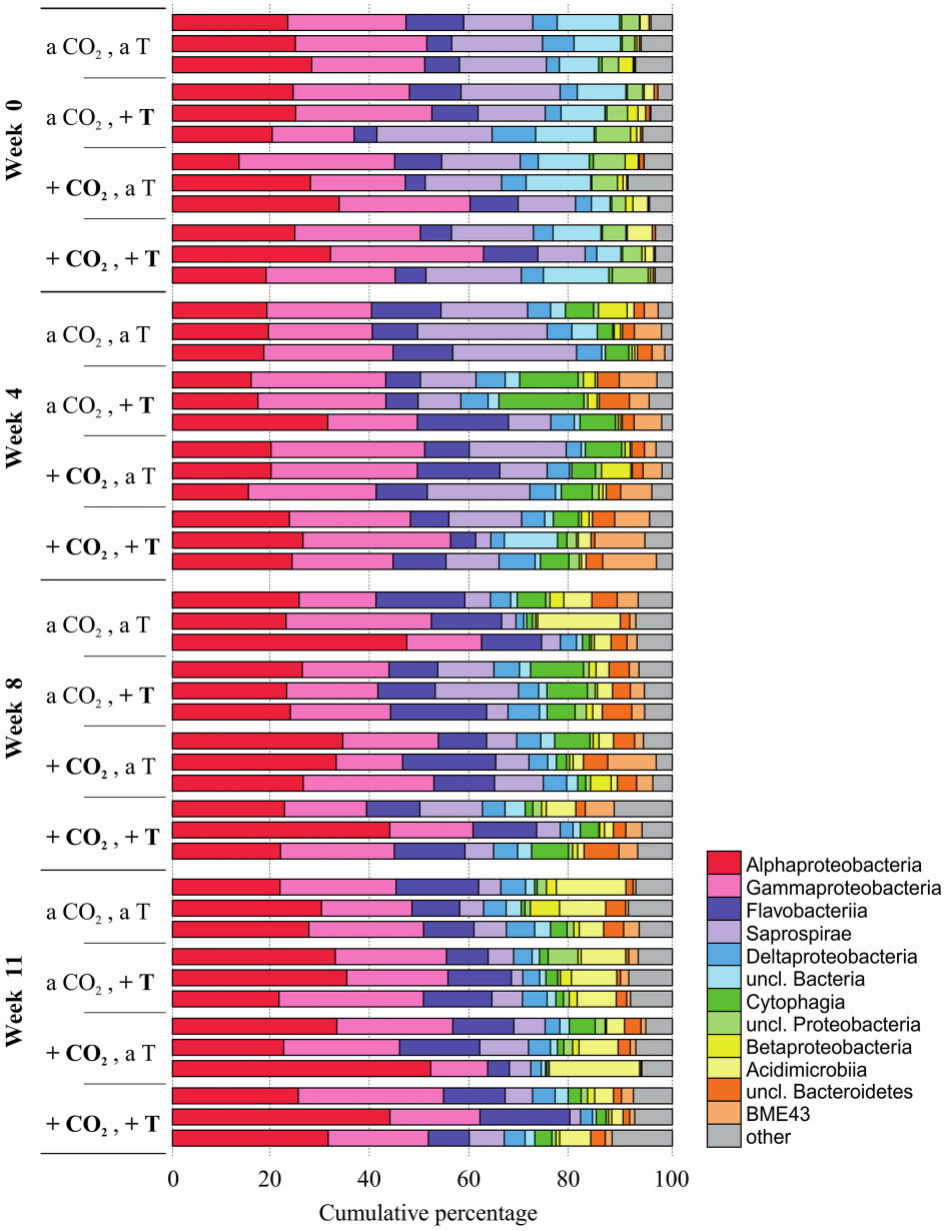
**Relative abundances (in cumulative percentage) of the most abundant epibacterial classes of *Fucus mytili***. Epibacterial community patterns are depicted for each sample (*n* = 3 per treatment) after 0, 4, 8, and 11 weeks of treatment in the benthic mesocosms at different conditions (see Section “Materials and Methods”; a, ambient; +, increased/elevated). Analysis is based on bacterial 16S rDNA (V1–V2) MiSeq amplicon sequencing. Low abundant classes (≤ 1%) were summarized as “other.”

In every week and treatment, the biofilm attached to the *F. mytili* surface was generally dominated by (in descending order) *Proteobacteria* (mainly *Alpha-* and *Gamma-*, fewer *Delta-*, and *Betaproteobacteria*), *Bacteroidetes* (*Flavobacteria, Saprospirae, Cytophagia, BME43* cluster), and *Actinobacteria* (*Acidimicrobiia*; Figure 2). Epibacterial community composition was highly variable between *F. mytili* replicates. At ambient conditions, *Alpha-* and *Gammaproteobacteria* together comprised up to ~50% of the epibacterial community on *F. mytili*. About 25% of the community consisted of *Flavobacteriia* and *Saprospirae*.

*Alpha-, Gammaproteobacteria*, and *Flavobacteriia* accounted for a major proportion of the free-living bacterial community in the seawater (Figure S4). In contrast to *F. mytili* biofilm the variation between replicates of water samples was smaller. Concerning biofilm and water samples, respectively, a week-wise evaluation based on OTU level revealed a development over time with weeks 0, 4, 8, and 11, remarkably (*F* ≥ 2) differing in their community composition (Figure S5).

### Alpha diversity of *F. mytili* microbiota was much higher than in seawater

**Figure 3:**
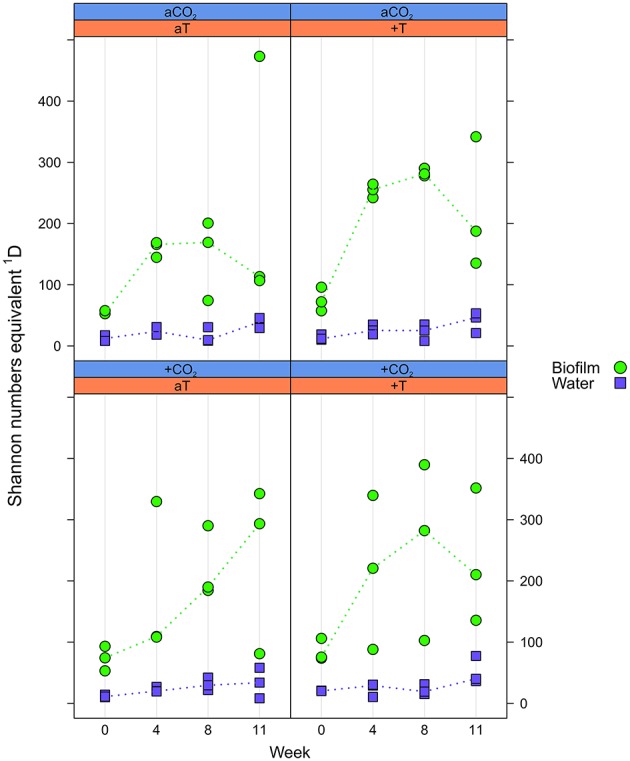
**Effective OTU richness**. Distribution of Shannon number equivalents ^1^D at OTU level concerning *Fucus mytili* biofilm (green circles) and water samples (blue diamonds) after 0, 4, 8, and 11 weeks of incubation in the benthic mesocosms under different conditions (see Section “Materials and Methods”; a, ambient; +, increased/elevated). Dots represent ^1^D of each single sample and dotted lines connect the medians (*n* = 3) between weeks.

Alpha diversity was expressed as effective OTU richness ^1^D (also known as Shannon numbers equivalents; Jost, 2006, 2007). In water samples, ^1^D showed small variation with markedly lower median values in the course of the experiment (max. ~50) compared to the *F. mytili* biofilm (up to median values of ~300; Figure 3). Unexpectedly, no clear differences in ^1^D were observed between *p*CO_2_ and temperature treatments compared to ambient controls, although biofilm samples showed a tendency of higher ^1^D in warm treatments (e.g., in week 8 the median value of warm treatments was ~290 vs. 160 under ambient control conditions). In general, diversity of *F. mytili* biofilm samples showed high variability with a tendency of higher ^1^D during the experiment compared to the initial time point (median value ~60).

### Elevated *p*CO_2_ affected all bacterial communities similarly

**Figure 4:**
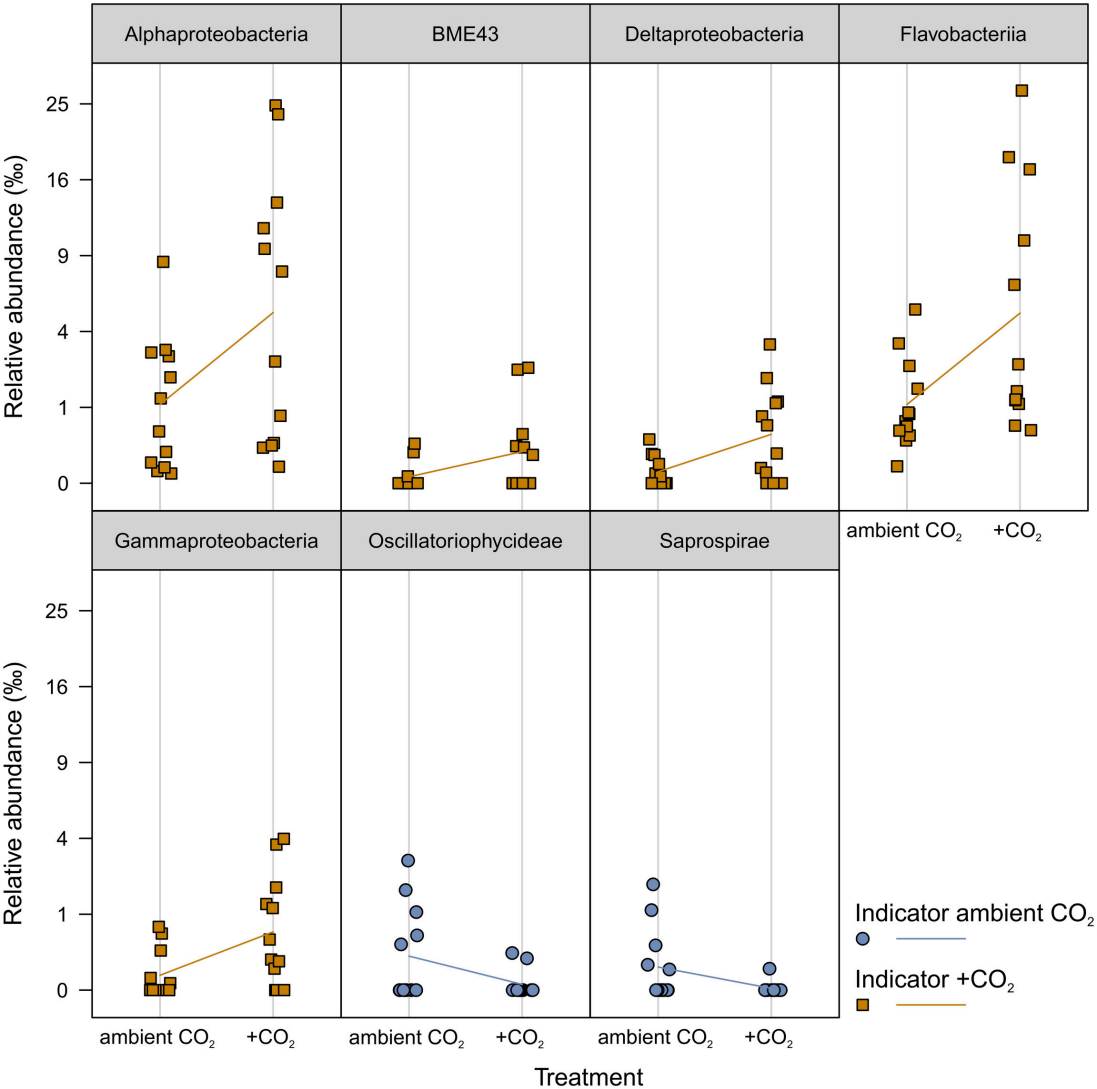
**Indicator OTUs (iOTUs) for *p*CO_2_ on all samples**. Relative abundances in %0 of single iOTUs for ambient (blue circles) and elevated (orange squares) *p*CO_2_ in week 8 summarized at class level (*q* ≤ 0.05) per sample (*n* = 12). Lines connect the mean relative abundances between both treatment levels. +CO_2_, elevated *p*CO_2_ treatment.

The *p*CO_2_ treatment resulted in a constant difference of ~0.2 pH units between ambient and elevated *p*CO_2_ levels; resulting in a delta of ~300 μatm *p*CO_2_ in the seawater (Figure S2). The inorganic nutrient concentrations were not affected by this *p*CO_2_ treatment (Figure S3). A weak time-dependent effect of *p*CO_2_ exclusively in week 8 [*F*_(1, 9)_ = 1.44, *p* ≈ 0.014] reshaped all bacterial communities in response to elevated *p*CO_2_ levels (Table S2). Further no interaction effect of *p*CO_2_ with temperature was observed. The *p*CO_2_ effect in week 8 explained 1.1% of the total variance. Comparatively few OTUs were affected by *p*CO_2_. Indicator OTUs (iOTUs) for elevated *p*CO_2_ (+CO_2_) belonged to the classes *Flavobacteriia, BME43, Alpha-, Delta-*, and *Gammaproteobacteria*, whereas iOTUs for ambient *p*CO_2_ (aCO_2_) are members of the *Oscillatoriophycideae* and *Saprospirae* (Figure 4). Highest differences in relative abundances due to +CO_2_ treatment were found among *Flavobacteriia* and *Alphaproteobacteria*, with noticeable iOTUs for +CO_2_ belonging to the genera *Pseudoruegeria* (OTU #7461, c_*Alphaproteobacteria*) and *Sediminicola* (OTU #1326, c_*Flavobacteriia*) with 0.38 and 2.79%0 in +CO_2_ compared to 0.01 and 0.38%0 in aCO_2_, respectively.

### Increased temperature affected the sample types differently

The temperature treatment resulted in a constant difference of ~5°C above ambient seawater temperature, with temperature naturally increasing during the course of the experiment (Figure S2). Inorganic nutrients were not affected by the temperature treatment (Figure S3). The microbiota significantly changed due to the temperature effect in weeks 4, 8, and 11, depending on the sample type (Biofilm:Temp:Week interaction *F* = 1.66, *p* = 0.006 and Water:Temp:Week interaction *F* = 1.66, *p* = 0.015; Table S2).

Epibacterial communities on *F. mytili* were significantly affected by temperature in weeks 4, 8, and 11 (*p* = 0.007, *p* = 0.002, and *p* = 0.001, respectively). The temperature effect on biofilm in weeks 4, 8, and 11 explained 10, 7, and 10% of total variance, respectively. Bacteria of the surrounding water column were significantly affected by temperature in weeks 4 and 8 (*p* = 0.004 and *p* = 0.008, respectively), while in week 11 there was only a trend detectable (*p* = 0.074). The temperature effect on water communities in weeks 4, 8, and 11 explained 18, 9, and 5 % of total variance, respectively.

**Figure 5:**
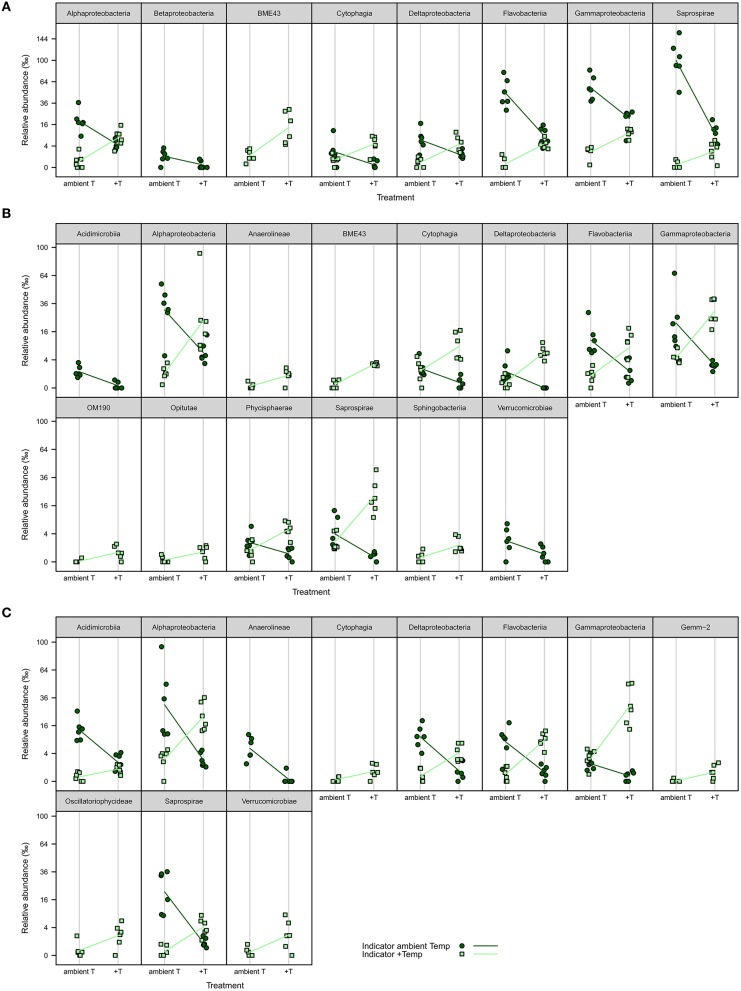
**iOTUs for temperature on *Fucus mytili***. Relative abundances in %0 of single iOTUs for ambient (dark green circles) and increased (light green squares) temperature on *F. mytili* in **(A)** week 4, **(B)** week 8, and **(C)** week 11 summarized at class level (*q* ≤ 0.05) per sample (*n* = 6). Lines connect the mean relative abundances between both treatment levels. +T, increased temperature treatment.

iOTUs for temperature effects on *F. mytili* biofilms in weeks 4, 8, and 11 belonged to 17 bacterial classes: *Alpha-, Beta-, Gamma-, Deltaproteobacteria, BME43, Cytophagia, Flavobacteriia, Saprospirae, Acidimicrobiia, Anaerolineae, OM190, Opitutae, Phycisphaerae, Sphingobacteriia, Verrucomicrobiae, Gemm-2*, and *Oscillatoriophycideae* (Figure 5). Most classes comprised iOTUs for both temperature levels, meaning that members of the same class were differently affected by temperature. In particular, iOTUs of *Alpha-, Delta-* and *Gammaproteobacteria* were present in each week and showed remarkably high differences in relative abundance between both treatment levels: Within *Alphaproteobacteria* most iOTUs belonged to the families *Rhodobacteraceae* and *Hyphomonadaceae*, and were found in both temperature levels. Among *Rhodobacteraceae, Octadecabacter antarcticus* (OTU#79) was an iOTU for ambient temperature (aT) with 4.72%0 mean relative abundance in week 11 compared to 0.46%0 at increased temperature (+T).

Most iOTUs within *Deltaproteobacteria* were members of the orders *Myxococcales* and *Bdellovibrionales*. Although both orders varied between aT and +T, *Myxococcales* (family *Nannocystaceae*) were more common at +T and *Bdellovibrionales* (family *Bacteriovoraceae*) more often at aT.

Within *Gammaproteobacteria*, iOTUs for both temperature levels belonged to the orders *Alteromonadales, Thiotrichales, Legionellales* and *Vibrionales*. In week 8, iOTUs for +T were assigned to family *Coxiellaceae* (OTU#1164/2823, 2.02%0 +T vs. 0.05%0 aT) and for aT to genus *Shewanella* (OTU#186, 1.70%0 +T vs. 0.00%0 aT). In week 11, iOTUs for +T belonged to family *Piscirickettsiaceae* (OTU#215/360/525/562/5390, 2.78%0 +T vs. 0.47%0 aT) and genus *Vibrio* (OTU#204, 3.33%0 +T vs. 0.02%0 aT), whereas an iOTU for aT belonged to *Candidatus Endobugula* (OTU#2952, 0.19%0 +T vs. 0.81%0 aT). The relative abundance of gammaproteobacterial iOTUs for +T remarkably increased from weeks 4–11, with a concurrent decrease of gammaproteobacterial iOTUs for aT.

**Figure 6:**
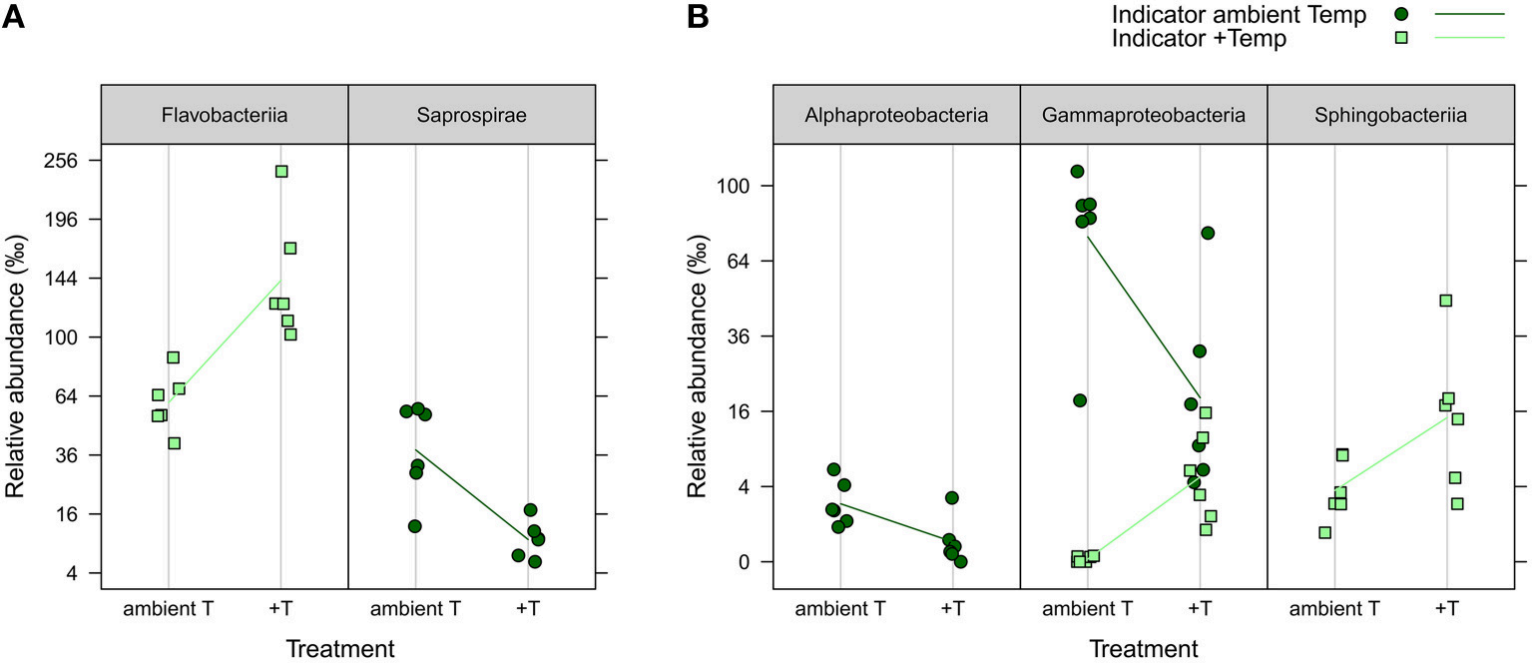
**iOTUs for temperature on seawater**. Relative abundances in %0 of single iOTUs for ambient (dark green circles) and increased (light green squares) temperature in the water column in **(A)** week 4 and **(B)** week 8 summarized at class level (*q* ≤ 0.05) per sample (*n* = 6). Lines connect the mean relative abundances between both treatment levels. +T, increased temperature treatment.

iOTUs for temperature effects on the water community in weeks 4 and 8 belonged to five bacterial classes: In week 4, iOTUs for +T belonged to *Flavobacteriia*, including members of the families *Flavobacteriaceae* and *Cryomorphaceae*, whereas iOTUs for aT belonged to the class *Saprospirae* (Figure 6). In week 8, OTUs of *Sphingobacteriia* were indicators for +T and OTUs of *Alphaproteobacteria* for aT. *Gammaproteobacteria* comprised iOTUs of both temperature levels, including iOTUs for +T of the family *Coxiellaceae* (OTU#1164, 4.96%0 +T vs. 0.01%0 aT) and the genus *Vibrio* (OTU#335, 1.34%0 +T vs. 0.00%0 aT), as well as iOTUs for aT of the genus *Candidatus Portiera* (OTU#6, 23.88%0 +T vs. 75.92%0 aT) and the family *Colwelliaceae* (OTU#193, 0.34%0 +T vs. 2.66%0 aT).

### Correlations between associated OTUs within the bacterial communities

OTU association network analysis revealed moderate to strong interactions between 25 OTUs (sorted by class-level taxonomy, indicator property and environmental distribution, respectively; see Figure S6 and Table S3). About half of the network-forming OTUs belong to *Alphaproteobacteria*, complemented by OTUs of *Gammaproteobacteria, Flavobacteriia, Betaproteobacteria, Saprospirae*, and the *BME43* cluster. Both positive and negative correlations between OTUs were detected, the latter between OTUs prevailing in different habitats (*F. mytili* biofilm vs. seawater) suggesting that relevant (i.e., moderate to strong) correlations between OTUs were mainly influenced by the environment and less by bacterial interactions.

### Increased temperature reduced relative biomass growth rates of the host *F. mytili*

**Figure 7:**
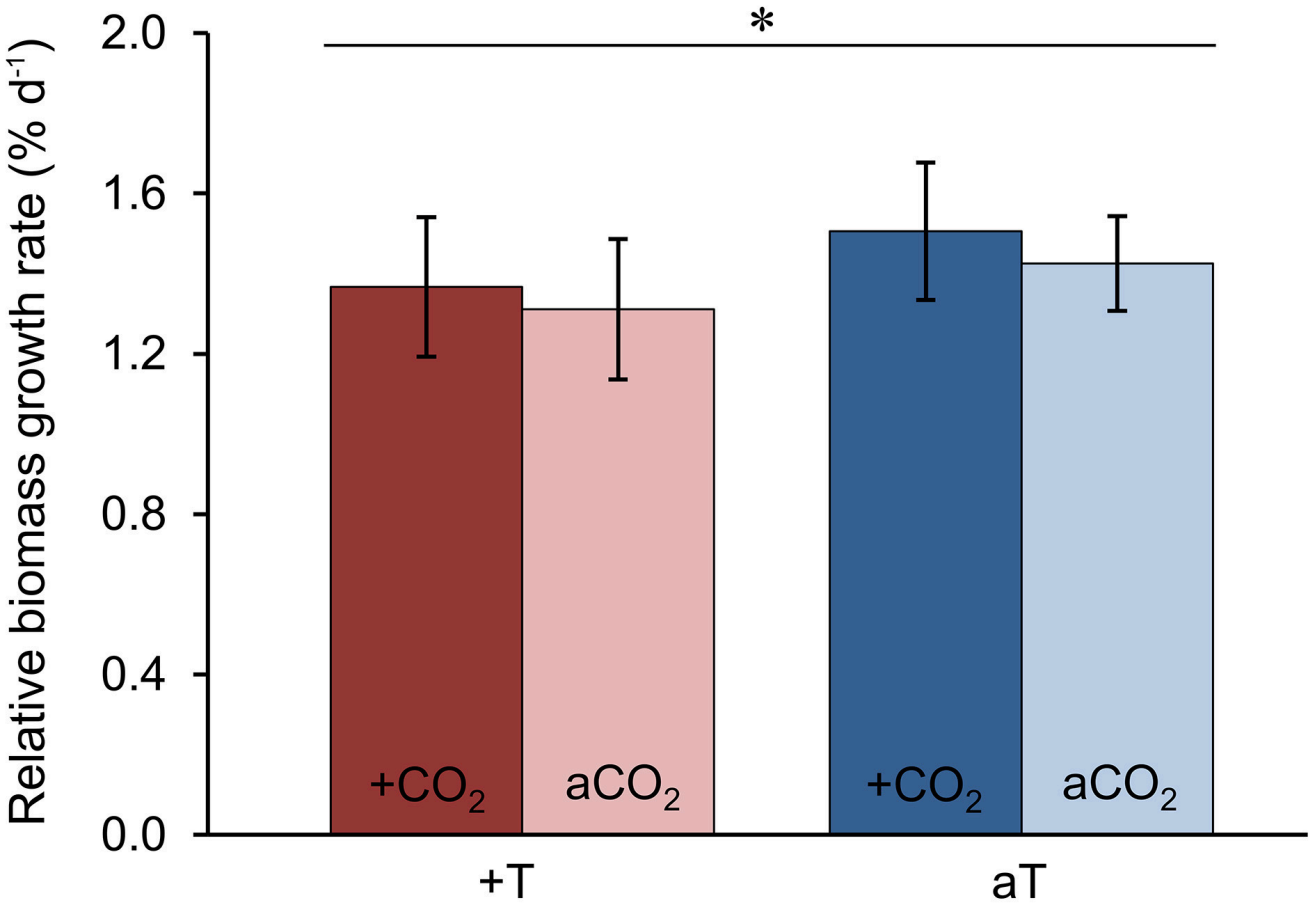
**Growth of *Fucus mytili***. Relative biomass growth rates in % d^−1^ of *F. mytili* algae after 11 weeks of incubation in the benthic mesocosms under different conditions (see Section “Materials and Methods”; a, ambient; +, increased/elevated). Mean values ± SD (*n* = 11) and asterisk (*p* < 0.05^*^) indicates significantly different changes of growth rates (two-way ANOVA) driven by temperature effect (*p* = 0.046^*^, *F* = 5.58, dfn = 1, dfd = 10).

In order to test the effects of increased temperature and elevated *p*CO_2_ levels on the physiological performance of the host-algae itself, the change in biomass of all *F. mytili* individuals (*n* = 11 per treatment) was analyzed (Figure 7). At ambient temperature, elevated *p*CO_2_ conditions slightly increased the growth rate of *F. mytili* (1.5 ± 0.3% d^−1^; mean ± SD; for definition of growth rate see Section“Materials and Methods”) compared to ambient *p*CO_2_ (1.4 ± 0.3% d^−1^), however this tendency was not significant (one-way ANOVA, *F* = 2.42, dfn = 3, dfd = 8, *p* = 0.14). Warming reduced growth rate of *F. mytili* significantly by 20% over the course of the experiment (two-way ANOVA, *F* = 5.58, dfn = 1, dfd = 10, *p* < 0.05). Growth rates of *F. mytili* under elevated *p*CO_2_ conditions at increased temperature tended to be higher (1.4 ± 0.4% d^−1^) compared to growth under warming alone (1.3 ± 0.3% d^−1^).

### Algal mannitol contents and C:N ratios were not affected by treatments

The carbon-to-nitrogen (C:N) ratio and the mannitol content were analyzed of *F. mytili* individuals used for biofilm sampling in the course of the entire experiment (*n* = 3 per treatment; see Table 1). The mannitol and carbon concentrations did not significantly change in the course of the experiment comparing initial and final values (one-way ANOVAs with *post hoc* Tukey's test, mannitol: *F* = 1.04, dfn = 4, dfd = 13, C: *F* = 1.56, dfn = 4, dfd = 13, *p* > 0.05). However, the C:N ratios obtained from samples at the end of the experiment were significantly higher for samples under all treatments compared to the initial values apparently due to significant decrease of the internal nitrogen concentration over time (one-way ANOVAs with *post hoc* Tukey's test, CN: *F* = 11.18, dfn = 4, dfd = 13, N: *F* = 1.56, dfn = 4, dfd = 13, *p* < 0.001). After 11 weeks of incubation, C:N ratios and mannitol concentrations did not significantly differ between all treatments (one-way ANOVAs with *post hoc* Tukey's test, CN: *F* = 0.53, dfn = 3, dfd = 8, mannitol: *F* = 0.51, dfn = 3, dfd = 8, *p* > 0.05).

**Table 1:** **Analysis of *Fucus mytili* tissue: carbon-to-nitrogen (C:N) ratios, C, N, and mannitol contents**.

	**C:N**	**C**	**N**	**Mannitol**
		**(% DW)**		
Initial	11.88 ±0.65a	34.68 ±0.49a	2.93 ±0.14a	13.92 ±2.18a
+T +CO_2_	23.42 ±5.60b	35.60 ±1.96a	1.57 ±0.33b	15.82 ±3.00a
+T aCO_2_	29.03 ±7.22b	36.31 ±0.78a	1.31 ±0.34b	22.12 ±9.87a
aT +CO_2_	25.80 ±5.29b	35.15 ±1.86a	1.40 ±0.26b	16.04 ±8.09a
aT aCO_2_	25.46 ±3.02b	36.42 ±0.89a	1.44 ±0.15b	17.21 ±5.74a

Data (in % DW, dry weight) of the initial physiological status of F. mytili in its native habitat (initial, n = 6) and after growing for 11 weeks (n = 3) under different temperature and pCO_2_ conditions (see Section “Materials and Methods”; a, ambient; +, increased/elevated). Values are means ± SD and different lowercase letters attached to values indicate significantly different means (p < 0.001; one-way ANOVA with Tukey's post hoc test).

## Discussion

### Microbiota of wadden sea *F. mytili* comparable to that of baltic *F. vesiculosus*

This first analysis of the epibacterial community composition of Wadden Sea *F. vesiculosus* forma *mytili* (*F. mytili*) demonstrated that the surface of *F. mytili* was colonized by *Proteobacteria* (*Alpha-, Gamma-, Delta-, Betaproteobacteria*), *Bacteroidetes* (*Flavobacteriia, Saprospirae, Cytophagia, BME43* cluster), and *Actinobacteria* (*Acidimicrobiia*). Two previous studies on the closely related macroalgal species, Baltic *F. vesiculosus*, also revealed *Proteobacteria* (*Alpha-, Gamma-, Delta-, Beta-*), *Bacteroidetes*, and *Actinobacteria* to be the most abundant phyla (Stratil et al., 2013, 2014). The latter was obtained from 16S rDNA V1-V2 amplicon pyrosequencing (454 Roche), hence offering a reliable comparison to our MiSeq amplicon data on *F. mytili* biofilms, in particular when restricted on the most abundant phyla or classes. The similarities between the epibacterial community compositions of North Sea *F. mytili* and Baltic *F. vesiculosus* was an unexpected finding, since the respective habitats highly differ in environmental conditions (e.g., salinity, nutrient concentrations and tidal range conditions). Thus the observed similarity of the epibacterial communities might be due to close genetic relationship of both hosts (Coyer et al., 2006) and might indicate similar host mechanisms to shape surface biofilm establishment. Our findings are in agreement with a comparative 16S rDNA cloning study of six macroalgal species (including *F. vesiculosus*) occurring in the Baltic and North Sea, respectively, which indicated less differences in epibacterial community composition between habitats than between different host species, moreover, the biofilms were more similar on closely related host species (Lachnit et al., 2009).

Although *Planctomycetes* are often reported to occur on macroalgae in remarkably high abundances (Bondoso et al., 2014; Lage and Bondoso, 2014), we rarely detected this phylum on *F. mytili* (e.g., class *Phycisphaerae* < 0.2% on average per week). However, the respective studies of those reports were mostly based on culture-based analysis (Lage and Bondoso, 2011) and thus do not necessarily reflect natural bacterial abundances. In agreement with our current sequencing results, Stratil and colleagues detected no *Planctomycetes* on Baltic *F. vesiculosus* also using a high-throughput sequencing approach (Stratil et al., 2013, 2014). Nonetheless, using 16S rDNA cloning and Sanger sequencing, we previously detected *Planctomycetes* on Baltic macroalgae, but simultaneously showed high variation in relative abundances of *Planctomycetes* between seasons/years and different macroalgae (Lachnit et al., 2011). Interestingly, Bengtsson et al. (2012) employed 16S amplicon pyrosequencing and found *Planctomycetes* to be the most abundant phylum (up to 56% of sequence reads) attached to the kelp *Laminaria hyperborea*. This finding again is in agreement with the already mentioned more general observation of Lachnit et al. (2009), that rather the host species than the geographic location determines the epibacterial community on macroalgae.

### Impact of elevated *p*CO_2_ on bacterial communities

Although the *p*CO_2_ treatment was successfully applied to the Sylt mesocosms, the effect on the bacterial communities attached to the algal surface and the planktonic ones was rather weak. This might be due to the fact that the majority of all environmental bacteria are known to have broader growth ranges with pH optima between pH 6 and 8 (Clark et al., 2009), thus they might be able to adapt to rapidly changing pH fluctuations. Moreover, it has been hypothesized by Mu and Moreau (2015) that biofilms exhibit larger tolerance to CO_2_ stress, which might explain the limited impact of the *p*CO_2_ treatment on the *F. mytili* biofilm community.

Nevertheless, the *p*CO_2_ effect was significant and we identified indicator OTUs (iOTUs): iOTUs of the genera *Pseudoruegeria* and *Sediminicola* showed higher relative abundance at elevated *p*CO_2_ levels. The fact that they are usually found at tidal flats (Jung et al., 2010) and deep-sea marine sediments (Hwang et al., 2015) where *p*CO_2_ is high due to organic matter decomposition, respectively, let us assume a—albeit slight—shift toward bacterial groups that are more adapted to higher *p*CO_2_ levels.

In contrast, in a previous microcosm study the CO_2_ effect appeared to be much more pronounced (Krause et al., 2012), however, these results are based on laboratory incubation scale and different sequencing techniques were used. This points to the general difficulty that the comparison of datasets obtained by different sequencing approaches are strongly limited due to a large variety of factors: In particular due to the chosen sequencing technology (e.g., Sanger sequencing, 454 Roche pyrosequencing, or Illumina MiSeq/HiSeq sequencing) and the sequenced region (e.g., full-length 16S rDNA or specific hypervariable regions of the 16S rDNA).

Overall our data suggested a *p*CO_2_ effect weaker than expected from recent literature. Previous studies with strong *p*CO_2_ effects refer to natural CO_2_-rich sites with long-term exposure of bacteria to high *p*CO_2_ (Oppermann et al., 2010), or experiments with overestimated *p*CO_2_ values up to 3000 μatm (Endres et al., 2014). Both do not reflect realistic future climate scenarios, as simulated in our mesocosm approach representing a near-natural approach with realistic evaluation of how marine bacterial communities could respond to future changes in seawater temperature and *p*CO_2_.

### Increased temperature affected both biofilm and water bacteria

Although most microorganisms are able to grow within a broad range of temperature (Clark et al., 2009), the temperature effect was markedly stronger than the *p*CO_2_ effect. Among epibacterial communities of *F. mytili*, we identified remarkably more temperature-related iOTUs than among seawater bacterial communities. Attached to the surface of *F. mytili*, an interesting iOTU for ambient temperature was *O. antarcticus*, known as cold-adapted species originally isolated from Antarctic Sea ice and water (Gosink et al., 1997). Additionally, the genera *Shewanella* and *C. Endobugula* were found to be more abundant at ambient temperature. They are known to be associated with fish tissue (Gram and Melchiorsen, 1996) and described as bacterial symbiont of *Bryozoa* (Lim and Haygood, 2004), respectively, thus seem to be related to healthy biofilms on a broad range of marine hosts. On the other hand, iOTUs of *Nannocystaceae* were indicators for increased temperature. Some members of this family were reported to degrade complex macromolecules and to lyse microorganisms (Garcia and Müller, 2014). Interestingly, we found several iOTUs associated with pathogenic features to be more abundant at increased temperature: The families *Coxiellaceae* (including pathogenic parasites; Lory, 2014) and *Piscirickettsiaceae* (including fish pathogens; Fryer and Hedrick, 2003), as well as the genus *Vibrio*. The latter comprises several thermophilic species associated with aquatic animal diseases like *V. harveyi* which is a pathogen of aquatic animals (Austin and Zhang, 2006). In agreement with our findings, Vezzulli and colleagues reported that temperature promotes *Vibrio* growth in the aquatic environment, supported by long-term data from the North Sea (Vezzulli et al., 2012, 2013).

In the water column increased temperature also led to significant shifts in community patterns. Remarkably, *Gammaproteobacteria* include iOTUs for both increased and ambient temperature, respectively. While typical marine members of the orders *Oceanospirillales* (genus *C. Portiera*) and *Alteromonadales* (family *Colwelliaceae*) were temperature-sensitive, some iOTUs of the orders *Legionellales* (family *Coxiellaceae*), and *Vibrionales* (genus *Vibrio*) increased in abundance with higher seawater temperature. In the latter case, *Coxiellaceae* and *Vibrio* include some parasites and pathogenic species, respectively (Austin and Zhang, 2006; Lory, 2014).

Overall, the increase in seawater temperature appeared to favor potentially pathogenic species over common and widespread bacteria. At increased seawater temperatures, our data showed a correlation between higher abundances of potentially pathogenic bacteria and reduced *F. mytili* biomass formation. Probably, thermally stressed *F. mytili* individuals might also exhibit reduced defense mechanisms, thus were more susceptible to infections.

### Bacteria-bacteria interactions described by OTU association network

Using network analysis, most associations were found between alphaproteobacterial OTUs. Basically, *Alphaproteobacteria* represented the most abundant class in both *F. mytili* biofilm and seawater samples, respectively, indicating that abundant bacteria have high impact on microbial structure and function (Lupatini et al., 2014). Positive correlations between OTUs suggest mutualistic interactions, while negative correlations might suggest a competition between bacteria (Steele et al., 2011). However, our network analysis showed negative correlations between OTUs to be due to environmental preferences (*F. mytili* surface or water column; Figure S6C).

Two typical water-associated OTUs, OTU#2 (*Flavobacteriaceae*, g_*Sediminicola*), and OTU#4 (*Cryomorphaceae*) were found to be strongly positively correlated with OTU#3 (*Rhodobacteraceae*, g_*Octadecabacter*), also predominantly found in water. Interestingly, while the former two OTUs were indicators for increased temperature in week 4 in water, OTU#3 was an indicator for ambient temperature in week 4 on *F. mytili*. This counterintuitive result becomes plausible if one keeps in mind that physiological status and activities can differ between surface-associated and free-living cells of the same taxon (Dang and Lovell, 2016). Thus, as part of the *F. mytili* biofilm OTU#3 may well prefer higher temperatures than it does as free-living cells.

We found *Bdellovibrio* to be an indicator for ambient temperature levels on *F. mytili* (week 11). However, no potential predatory OTUs (e.g., BALOs; Williams and Piñeiro, 2007) were found in the network, because they were not among the OTUs consistently present across the majority of samples. Thus, our dataset does not provide evidence for predatory bacteria to be involved in reshaping the communities as reviewed by Dang and Lovell (2016). However, important impacts of predatory bacteria on bacterial community restructuring might occur during other seasons.

### *F. mytili* growth rates but not physiological features were affected by treatments

Our data showed significantly reduced growth rates of *F. mytili* at increased temperatures often reaching daily maxima of 24°C, suggesting that increased seawater temperature reduced biomass formation of this macroalga. Recently, Graiff et al. (2015a) described an inhibitory temperature effect on biomass growth rates of Baltic *F. vesiculosus* in most seasons in a comparable experimental design. Here, 24°C was a thermal threshold with biomass production of Baltic *F. vesiculosus* starting to stagnate, and above 24°C growth rates declined probably because physiological cell processes were affected (Graiff et al., 2015b). Elevated *p*CO_2_ appeared to enhance growth of *F. mytili* even under warmed conditions. The provision of increased amounts of dissolved inorganic carbon (DIC) may have increased—antagonistically to warming—the performance of *F. mytili*. This finding is consistent with Graiff et al. (2015a), who examined the combined effects of *p*CO_2_ and temperature on Baltic *F. vesiculosus* under similar conditions. Mitigating effects of enhanced *p*CO_2_ on growth performance of thermally stressed algae were previously reported, e.g., for the red alga *Chondrus crispus* (Sarker et al., 2013).

Contrary to the growth rates of *F. mytili*, C:N ratios and mannitol concentrations were not significantly affected by the applied treatments. This is in agreement with findings on Baltic *F. vesiculosus* studied under natural conditions (Lehvo et al., 2001) as well as under similar temperature and *p*CO_2_ treatments in the Kiel Outdoor Benthocosms (Graiff et al., 2015a). Further evidence is provided by Gutow et al. (2014) detecting no significant *p*CO_2_ effect on C:N ratios of North Sea *F. vesiculosus*. The C:N ratios of North Sea *F. mytili* were comparable to that found in Baltic *F. vesiculosus* during spring (Graiff et al., 2015a). Highest differences between initial (at the beginning of April) and final (at the end of June) nitrogen concentrations were typically found in spring for both *Fucus* species. These results suggest high internal nitrogen storage during winter to spring (before the spring bloom depleted the dissolved inorganic nitrogen of seawater) for stimulating rapid summer growth in nitrogen-depleted conditions (Lehvo et al., 2001). Thus, the increasing C:N ratios of *F. mytili* during our experiment resulted from decreasing nitrogen concentrations in the seawater (leading to nitrogen limitation due to the spring phytoplankton bloom) and depletion of the internal nitrogen storage of *F. mytili* due to investment into increased growth.

Mannitol represents a major end product of photosynthesis in brown algae (Phaeophyceae) and serves as energy source for growth and/or survival during the dark winter period (Dunton and Schell, 1986; Dunton and Jodwalis, 1988; Lehvo et al., 2001; Wiencke et al., 2009; Rousvoal et al., 2011; Gómez and Huovinen, 2012). Here, mannitol contents of *F. mytili* are very similar to those in Baltic *F. vesiculosus* (Graiff et al., 2015a), suggesting high mannitol accumulation in spring and summer for maintaining growth in winter months (Lehvo et al., 2001). The mannitol concentration in *F. mytili* did not change significantly over time, neither due to the applied treatments indicating that the photosynthesis was not influenced by increased temperature or elevated *p*CO_2_. Interestingly, both *Fucus* species showed highest variation in mannitol concentrations when exposed to increased temperature (+5°C, single factor treatment) during spring (Graiff et al., 2015a), probably due to individual phenotypic plasticity. Although the Wadden Sea *F. mytili* appeared to be as well adapted as the Baltic *F. vesiculosus* to the conditions of northern latitudes (Lehvo et al., 2001), we cannot rule out future *F. mytili* dying during the summer period when exposed to an increased seawater temperature by +5°C, as recently reported for Baltic *F. vesiculosus* (Graiff et al., 2015a). In the latter case, *F. mytili* would become the limiting factor with severe consequences for the marine ecosystem.

Overall we obtained evidence indicating that increasing seawater temperature as predicted for the future ocean might result in significant restructuring of the epibacterial community on *F. mytili*. Moreover, this might significantly increase the number of potentially pathogenic bacteria attached to the surface of the marine macroalgae, which ultimately will have consequences for the host-microbe interactions.

## Author contributions

BM designed the study, carried out sampling and sample processing, analyzed, and interpreted the data, and wrote the manuscript. SN performed the statistical analyses on the sequencing data. AG analyzed the algal tissue and performed statistics on all algae-related data. AP measured the water parameters and nutrients, and maintained technical support of the mesocosms. MF designed the MiSeq primer and supported the bioinformatical analysis. SK sequenced the amplicon samples. RS designed the experiment, took part in writing the manuscript, founded and supervised the work. All authors gave input to the manuscript.

### Conflict of interest statement

Conflict of Interest Statement: The authors declare that the research was conducted in the absence of any commercial or financial relationships that could be construed as a potential conflict of interest.

## References

[B1] Albrecht A. S. (1998). Soft bottom versus hard rock: community ecology of macroalgae on intertidal mussel beds in the Wadden Sea. J. Exp. Mar. Biol. Ecol. 229, 85–109. 10.1016/S0022-0981(98)00044-6

[B2] Austin B. Zhang X. H. (2006). Vibrio harveyi: a significant pathogen of marine vertebrates and invertebrates. Lett. Appl. Microbiol. 43, 119–124. 10.1111/j.1472-765X.2006.01989.x 16869892

[B3] Bengtsson M. M. Sjøtun K. Lanzén A. Ovreås L. (2012). Bacterial diversity in relation to secondary production and succession on surfaces of the kelp *Laminaria hyperborea*. ISME J. 6, 2188–2198. 10.1038/ismej.2012.67 22763650 PMC3505018

[B4] Benjamini Y. Hochberg Y. (1995). Controlling the false discovery rate - a practical and powerful approach to multiple testing. J. R. Stat. Soc. B Methodol. 57, 289–300.

[B5] Bondoso J. Balagué V. Gasol J. M. Lage O. M. (2014). Community composition of the Planctomycetes associated with different macroalgae. FEMS Microbiol. Ecol. 88, 445–456. 10.1111/1574-6941.12258 24266389

[B6] Bosch T. C. G. McFall-Ngai M. J. (2011). Metaorganisms as the new frontier. Zoology 114, 185–190. 10.1016/j.zool.2011.04.001 21737250 PMC3992624

[B7] Chen V. B. Davis I. W. Richardson D. C. (2009). KiNG (Kinemage, Next Generation): a versatile interactive molecular and scientific visualization program. Protein Sci. 18, 2403–2409. 10.1002/pro.250 19768809 PMC2788294

[B8] Clark D. P. Dunlap P. Madigan M. Martinko J. (2009). Brock Biology of Microorganisms. Beijing: Scientific Publisher.

[B9] Coyer J. A. Hoarau G. Oudot-Le Secq M. P. Stam W. T. Olsen J. L. (2006). A mtDNA-based phylogeny of the brown algal genus Fucus (Heterokontophyta; Phaeophyta). Mol. Phylogenet. Evol. 39, 209–222. 10.1016/j.ympev.2006.01.019 16495086

[B10] Csárdi G. Nepusz T. (2006). The Igraph Software Package for Complex Network Research. Kalamazoo, MI; Budapest: InterJournal Complex Systems, 1695.

[B11] Dang H. Lovell C. R. (2016). Microbial surface colonization and biofilm development in marine environments. Microbiol. Mol. Biol. Rev. 80, 91–138. 10.1128/MMBR.00037-15 26700108 PMC4711185

[B12] De Cáceres M. Legendre P. (2009). Associations between species and groups of sites: indices and statistical inference. Ecology 90, 3566–3574. 10.1890/08-1823.1 20120823

[B13] Dickson A. G. (1984). Ph scales and proton-transfer reactions in saline media such as sea-water. Geochim. Cosmochim. Acta 48, 2299–2308. 10.1016/0016-7037(84)90225-4

[B14] Dittami S. M. Gobet A. Duboscq-Bidot L. Perennou M. Corre E. Frioux C. (2015). Microbiomes impact algal acclimation: the example of a freshwater strain of ectocarpus. Eur. J. Phycol. 50, 44–44.

[B15] Dunton K. H. Jodwalis C. M. (1988). Photosynthetic performance of laminaria-solidungula measured *insitu* in the alaskan high arctic. Mar. Biol. 98, 277–285. 10.1007/BF00391206

[B16] Dunton K. H. Schell D. M. (1986). Seasonal carbon budget and growth of laminaria-solidungula in the alaskan high arctic. Mar. Ecol. Prog. Ser. 31, 57–66. 10.3354/meps031057

[B17] Edgar R. C. Haas B. J. Clemente J. C. Quince C. Knight R. (2011). UCHIME improves sensitivity and speed of chimera detection. Bioinformatics 27, 2194–2200. 10.1093/bioinformatics/btr381 21700674 PMC3150044

[B18] Egan S. Harder T. Burke C. Steinberg P. Kjelleberg S. Thomas T. (2013). The seaweed holobiont: understanding seaweed-bacteria interactions. FEMS Microbiol. Rev. 37, 462–476. 10.1111/1574-6976.12011 23157386

[B19] Endres S. Galgani L. Riebesell U. Schulz K. G. Engel A. (2014). Stimulated bacterial growth under elevated pCO(2): results from an off-shore mesocosm study. PLoS ONE 9:e99228 10.1371/journal.pone.0099228 24941307 PMC4062391

[B20] Fortunato C. S. Eiler A. Herfort L. Needoba J. A. Peterson T. D. Crump B. C. (2013). Determining indicator taxa across spatial and seasonal gradients in the Columbia River coastal margin. ISME J. 7, 1899–1911. 10.1038/ismej.2013.79 23719153 PMC3965310

[B21] Fryer J. L. Hedrick R. P. (2003). Piscirickettsia salmonis: a Gram-negative intracellular bacterial pathogen of fish. J. Fish Dis. 26, 251–262. 10.1046/j.1365-2761.2003.00460.x 12962234

[B22] Garcia R. Müller R. (2014). The Family Nannocystaceae, in The Prokaryotes, eds Rosenberg E. Delong E. Lory S. Stackebrandt E. Thompson F. (Berlin; Heidelberg: Springer), 213–229.

[B23] Goecke F. Labes A. Wiese J. Imhoff J. F. (2010). Chemical interactions between marine macroalgae and bacteria. Mar. Ecol. Prog. Ser. 409, 267–299. 10.3354/meps08607

[B24] Gómez I. Huovinen P. (2012). Morpho-functionality of carbon metabolism in seaweeds, in Seaweed Biology, eds Wiencke C. Bischof K. (Berlin; Heidelberg: Springer), 25–46.

[B25] Gosink J. J. Herwig R. P. Staley J. T. (1997). Octadecabacter arcticus gen nov, sp nov, and *O. antarcticus, sp nov, nonpigmented, psychrophilic gas vacuolate bacteria from polar sea ice and water*. Syst. Appl. Microbiol. 20, 356–365. 10.1016/S0723-2020(97)80003-3

[B26] Graiff A. Bartsch I. Ruth W. Wahl M. Karsten U. (2015a). Season exerts differential effects of ocean acidification and warming on growth and carbon metabolism of the seaweed *Fucus vesiculosus* in the western Baltic Sea. Front. Mar. Sci. 2:112 10.3389/fmars.2015.00112

[B27] Graiff A. Liesner D. Karsten U. Bartsch I. (2015b). Temperature tolerance of western Baltic Sea *Fucus vesiculosus* - growth, photosynthesis and survival. J. Exp. Mar. Biol. Ecol. 471, 8–16. 10.1016/j.jembe.2015.05.009

[B28] Gram L. Melchiorsen J. (1996). Interaction between fish spoilage bacteria *Pseudomonas* sp. and Shewanella putrefaciens in fish extracts and on fish tissue. J. Appl. Bacteriol. 80, 589–595. 10.1111/j.1365-2672.1996.tb03262.x 8698659

[B29] Gutow L. Rahman M. M. Bartl K. Saborowski R. Bartsch I. Wiencke C. (2014). Ocean acidification affects growth but not nutritional quality of the seaweed *Fucus vesiculosus* (*Phaeophyceae, Fucales*). J. Exp. Mar. Biol. Ecol. 453, 84–90. 10.1016/j.jembe.2014.01.005

[B30] Hellio C. Bremer G. Pons A. M. Le Gal Y. Bourgougnon N. (2000). Inhibition of the development of microorganisms (bacteria and fungi) by extracts of marine algae from Brittany, France. Appl. Microbiol. Biotechnol. 54, 543–549. 10.1007/s002530000413 11092630

[B31] Hwang C. Y. Lee I. Cho Y. R. Lee Y. M. Jung Y. J. Baek K.. (2015). Sediminicola arcticus sp nov., a psychrophilic bacterium isolated from deep-sea sediment, and emended description of the genus Sediminicola. Int. J. Syst. Evol. Microbiol. 65, 1567–1571. 10.1099/ijs.0.000138 25713047

[B32] IPCC (2013). Climate Change 2013: The Physical Science Basis. Contribution of Working Group I to the Fifth Assessment Report of the Intergovernmental Panel on Climate Change, eds Stocker T. F. Qin D. Plattner G.-K. Tignor M. Allen S. K. Boschung J. Nauels A. Xia Y. Bex V. Midgley P. M. (Cambridge, UK; New York, NY: Cambridge University Press), 1535.

[B33] IPCC, (2014). Climate Change 2014: Impacts, Adaptation, and Vulnerability. Part A: Global and Sectoral Aspects. Contribution of Working Group II to the Fifth Assessment Report of the Intergovernmental Panel on Climate Change, eds Field C. B. Barros V. R. Dokken D. J. Mach K. J. Mastrandrea M. D. Bilir T. E. Chatterjee M. Ebi K. L. Estrada Y. O. Genova R. C. Girma B. Kissel E. S. Levy A. N. MacCracken S. Mastrandrea P. R. White L. L. (Cambridge, UK; New York, NY: Cambridge University Press), 1132.

[B34] Jost L. (2006). Entropy and diversity. Oikos 113, 363–375. 10.1111/j.2006.0030-1299.14714.x

[B35] Jost L. (2007). Partitioning diversity into independent alpha and beta components. Ecology 88, 2427–2439. 10.1890/06-1736.1 18027744

[B36] Jung Y. T. Kim B. H. Oh T. K. Yoon J. H. (2010). Pseudoruegeria lutimaris sp nov., isolated from a tidal flat sediment, and emended description of the genus Pseudoruegeria. Int. J. Syst. Evol. Microbiol. 60, 1177–1181. 10.1099/ijs.0.015073-0 19667391

[B37] Karsten U. Thomas D. N. Weykam G. Daniel C. Kirst G. O. (1991). A simple and rapid method for extraction and separation of low-molecular-weight carbohydrates from macroalgae using high-performance liquid-chromatography. Plant Physiol. Biochem. 29, 373–378.

[B38] Kozich J. J. Westcott S. L. Baxter N. T. Highlander S. K. Schloss P. D. (2013). Development of a dual-index sequencing strategy and curation pipeline for analyzing amplicon sequence data on the miseq illumina sequencing platform. Appl. Environ. Microbiol. 79, 5112–5120. 10.1128/AEM.01043-13 23793624 PMC3753973

[B39] Krause E. Wichels A. Giménez L. Lunau M. Schilhabel M. B. Gerdts G. (2012). Small changes in pH have direct effects on marine bacterial community composition: a microcosm approach. PLoS ONE 7:e47035. 10.1371/journal.pone.0047035 23071704 PMC3469576

[B40] Kurtz Z. D. Müller C. L. Miraldi E. R. Littman D. R. Blaser M. J. Bonneau R. A. (2015). Sparse and compositionally robust inference of microbial ecological networks. PLoS Comput. Biol. 11:e1004226. 10.1371/journal.pcbi.1004226 25950956 PMC4423992

[B41] Lachnit T. Blumel M. Imhoff J. F. Wahl M. (2009). Specific epibacterial communities on macroalgae: phylogeny matters more than habitat. Aquat. Biol. 5, 181–186. 10.3354/ab00149

[B42] Lachnit T. Meske D. Wahl M. Harder T. Schmitz R. (2011). Epibacterial community patterns on marine macroalgae are host-specific but temporally variable. Environ. Microbiol. 13, 655–665. 10.1111/j.1462-2920.2010.02371.x 21078035

[B43] Lachnit T. Wahl M. Harder T. (2010). Isolated thallus-associated compounds from the macroalga *Fucus vesiculosus* mediate bacterial surface colonization in the field similar to that on the natural alga. Biofouling 26, 247–255. 10.1080/08927010903474189 20054721

[B44] Lage O. M. Bondoso J. (2011). Planctomycetes diversity associated with macroalgae. FEMS Microbiol. Ecol. 78, 366–375. 10.1111/j.1574-6941.2011.01168.x 21726244

[B45] Lage O. M. Bondoso J. (2014). Planctomycetes and rnacroalgae, a striking association. Front. Microbiol. 5:267. 10.3389/fmicb.2014.00267 24917860 PMC4042473

[B46] Langfeldt D. Neulinger S. C. Heuer W. Staufenbiel I. Kunzel S. Baines J. F.. (2014). Composition of microbial oral biofilms during maturation in young healthy adults. PLoS ONE 9:e87499. 10.1371/journal.pone.0087449 24503584 PMC3913613

[B47] Lehvo A. Back S. Kiirikki M. (2001). Growth of *Fucus vesiculosus* L. (Phaeophyta) in the northern Baltic proper: energy and nitrogen storage in seasonal environment. Botan. Mar. 44, 345–350. 10.1515/BOT.2001.044

[B48] Levitus S. Antonov J. I. Boyer T. P. Stephens C. (2000). Warming of the world ocean. Science 287, 2225–2229. 10.1126/science.287.5461.2225

[B49] Lim G. E. Haygood M. G. (2004). “*Candidatus endobugula* glebosa,” a specific bacterial symbiont of the marine bryozoan Bugula simplex. Appl. Environ. Microbiol. 70, 4921–4929. 10.1128/AEM.70.8.4921-4929.2004 15294832 PMC492373

[B50] Lory S. (2014). The Family Coxiellaceae, in The Prokaryotes, eds Rosenberg E. Delong E. Lory S. Stackebrandt E. Thompson F. (Berlin; Heidelberg: Springer), 197–198.

[B51] Löscher C. R. Fischer M. A. Neulinger S. C. Fiedler B. Philippi M. Schutte F. (2015). Hidden biosphere in an oxygen-deficient Atlantic open-ocean eddy: future implications of ocean deoxygenation on primary production in the eastern tropical North Atlantic. Biogeosciences 12, 7467–7482. 10.5194/bg-12-7467-2015

[B52] Lüning K. Yarish C. Kirkman H. (1990). Seaweeds: Their Environment, Biogeography, and Ecophysiology. New York, NY: John Wiley & Sons.

[B53] Lupatini M. Suleiman A. Jacques R. Antoniolli Z. Ferreira A. Kuramae E. E. (2014). Network topology reveal high connectance levels and few key microbial genera within soils. Front. Environ. Sci. 2:10 10.3389/fenvs.2014.00010

[B54] Martin M. Portetelle D. Michel G. Vandenbol M. (2014). Microorganisms living on macroalgae: diversity, interactions, and biotechnological applications. Appl. Microbiol. Biotechnol. 98, 2917–2935. 10.1007/s00253-014-5557-2 24562178

[B55] McFall-Ngai M. Hadfield M. G. Bosch T. C. G. Carey H. V. Domazet-Lošo T. Douglas A. E.. (2013). Animals in a bacterial world, a new imperative for the life sciences. Proc. Natl. Acad. Sci. U.S.A. 110, 3229–3236. 10.1073/pnas.1218525110 23391737 PMC3587249

[B56] Meinshausen N. Bühlmann P. (2006). High-dimensional graphs and variable selection with the lasso. Ann. Stat. 34, 1436–1462. 10.1214/009053606000000281

[B57] Mu A. Moreau J. W. (2015). The geomicrobiology of CO2 geosequestration: a focused review on prokaryotic community responses to field-scale CO2 injection. Front. Microbiol. 6:263. 10.3389/fmicb.2015.00263 25914677 PMC4391042

[B58] Nasrolahi A. Stratil S. B. Jacob K. J. Wahl M. (2012). A protective coat of microorganisms on macroalgae: inhibitory effects of bacterial biofilms and epibiotic microbial assemblages on barnacle attachment. FEMS Microbiol. Ecol. 81, 583–595. 10.1111/j.1574-6941.2012.01384.x 22486721

[B59] Nienburg W. (1932). Fucus Mytili spec. nov. Ber. d. Bot. Ges. 50a, 28–41.

[B60] Oksanen J. Blanchet F. G. Kindt R. Legendre P. Minchin P. R. O'hara R. B. (2015). Vegan: Community Ecology Package. R Package Version 2.4-0. Available online at: https://github.com/vegandevs/vegan

[B61] Oppermann B. I. Michaelis W. Blumenberg M. Frerichs J. Schulz H. M. Schippers A. (2010). Soil microbial community changes as a result of long-term exposure to a natural CO2 vent. Geochim. Cosmochim. Acta 74, 2697–2716. 10.1016/j.gca.2010.02.006

[B62] Pansch A. Winde V. Asmus R. Asmus H. (2016). Tidal benthic mesocosms simulating future climate change scenarios in the field of marine ecology. Limnol. Oceanogr. [Epub ahead of print]. 10.1002/lom3.10086

[B63] Pierrot D. Lewis E. Wallace D. (2006). MS Excel Program Developed for CO2 System Calculations. ORNL/CDIAC-105a. Oak Ridge, TN: Carbon Dioxide Information Analysis Center, Oak Ridge National Laboratory, US Department of Energy.

[B64] Pruesse E. Quast C. Knittel K. Fuchs B. M. Ludwig W. G. Peplies J.. (2007). SILVA: a comprehensive online resource for quality checked and aligned ribosomal RNA sequence data compatible with ARB. Nucleic Acids Res. 35, 7188–7196. 10.1093/nar/gkm864 17947321 PMC2175337

[B65] Raven J. Caldeira K. Elderfield H. Hoegh-Guldberg O. Liss P. Riebesell U. (2005). Ocean Acidification Due to Increasing Atmospheric Carbon Dioxide. The Royal Society.

[B66] R Core Team (2015). R: A Language and Environment for Statistical Computing, Vienna. Available online at: URL http://www.R-project.org

[B67] Richardson D. C. Richardson J. S. (1992). The kinemage: a tool for scientific communication. Protein Sci. 1, 3–9. 10.1002/pro.5560010102 1304880 PMC2142077

[B68] Rousvoal S. Groisillier A. Dittami S. M. Michel G. Boyen C. Tonon T. (2011). Mannitol-1-phosphate dehydrogenase activity in Ectocarpus siliculosus, a key role for mannitol synthesis in brown algae. Planta 233, 261–273. 10.1007/s00425-010-1295-6 20981555

[B69] Sarkar D. (2008). Lattice: Multivariate Data Visualization with R. New York, NY: Springer Science & Business Media.

[B70] Sarker M. Y. Bartsch I. Olischläger M. Gutow L. Wiencke C. (2013). Combined effects of CO2, temperature, irradiance and time on the physiological performance of *Chondrus crispus* (Rhodophyta). Botan. Mar. 56, 63–74. 10.1515/bot-2012-0143

[B71] Schloss P. D. Westcott S. L. Ryabin T. Hall J. R. Hartmann M. Hollister E. B.. (2009). Introducing mothur: open-source, platform-independent, community-supported software for describing and comparing microbial communities. Appl. Environ. Microbiol. 75, 7537–7541. 10.1128/AEM.01541-09 19801464 PMC2786419

[B72] Schories D. Albrecht A. Lotze H. (1997). Historical changes and inventory of macroalgae from Konigshafen Bay in the northern Wadden Sea. Helgolander Meeresuntersuchungen 51, 321–341. 10.1007/BF02908718

[B73] Simpson G. L. (2015). Permute: Functions for Generating Restricted Permutations of Data. R Package Version 0.8-4. Available onlne at: http://CRAN.R-project.org/package=permute

[B74] Singh R. P. Reddy C. R. K. (2014). Seaweed-microbial interactions: key functions of seaweed-associated bacteria. FEMS Microbiol. Ecol. 88, 213–230. 10.1111/1574-6941.12297 24512602

[B75] Steele J. A. Countway P. D. Xia L. Vigil P. D. Beman J. M. Kim D. Y.. (2011). Marine bacterial, archaeal and protistan association networks reveal ecological linkages. ISME J. 5, 1414–1425. 10.1038/ismej.2011.24 21430787 PMC3160682

[B76] Stewart R. I. A. Dossena M. Bohan D. A. Jeppesen E. Kordas R. L. Ledger M. E. (2013). Mesocosm experiments as a tool for ecological climate-change research. Adv. Ecol. Res. 48, 71–181. 10.1016/b978-0-12-417199-2.00002-1

[B77] Stratil S. B. Neulinger S. C. Knecht H. Friedrichs A. K. Wahl M. (2013). Temperature-driven shifts in the epibiotic bacterial community composition of the brown macroalga *Fucus vesiculosus*. Microbiologyopen 2, 338–349. 10.1002/mbo3.79 23568841 PMC3633357

[B78] Stratil S. B. Neulinger S. C. Knecht H. Friedrichs A. K. Wahl M. (2014). Salinity affects compositional traits of epibacterial communities on the brown macroalga *Fucus vesiculosus*. FEMS Microbiol. Ecol. 88, 272–279. 10.1111/1574-6941.12292 24490649

[B79] Verardo D. J. Froelich P. N. McIntyre A. (1990). Determination of organic-carbon and nitrogen in marine-sediments using the Carlo-Erba-Na-1500 analyzer. Deep Sea Res. A Oceanogr. Res. Papers 37, 157–165. 10.1016/0198-0149(90)90034-S

[B80] Vezzulli L. Brettar I. Pezzati E. Reid P. C. Colwell R. R. Hofle M. G.. (2012). Long-term effects of ocean warming on the prokaryotic community: evidence from the vibrios. ISME J. 6, 21–30. 10.1038/ismej.2011.89 21753799 PMC3246245

[B81] Vezzulli L. Colwell R. R. Pruzzo C. (2013). Ocean warming and spread of pathogenic vibrios in the aquatic environment. Microb. Ecol. 65, 817–825. 10.1007/s00248-012-0163-2 23280498

[B82] Wahl M. Buchholz B. Winde V. Golomb D. Guy-Haim T. Müller J. (2015). A mesocosm concept for the simulation of near-natural shallow underwater climates: the Kiel Outdoor Benthocosms (KOB). Limnol. Oceanogr. 13, 651–663. 10.1002/lom3.10055

[B83] Wang Q. Garrity G. M. Tiedje J. M. Cole J. R. (2007). Naive Bayesian classifier for rapid assignment of rRNA sequences into the new bacterial taxonomy. Appl. Environ. Microbiol. 73, 5261–5267. 10.1128/AEM.00062-07 17586664 PMC1950982

[B84] Weinberger F. (2007). Pathogen-induced defense and innate immunity in macroalgae. Biol. Bull. 213, 290–302. 10.2307/25066646 18083968

[B85] Wichard T. (2015). Exploring bacteria-induced growth and morphogenesis in the green macroalga order Ulvales (Chlorophyta). Front. Plant Sci. 6:86. 10.3389/fpls.2015.00086 25784916 PMC4347444

[B86] Wiencke C. Gomez I. Dunton K. (2009). Phenology and seasonal physiological performance of polar seaweeds. Botan. Mar. 52, 585–592. 10.1515/BOT.2009.078

[B87] Williams H. N. Piñeiro S. (2007). Ecology of the predatory bdellovibrio and like organisms, in Predatory Prokaryotes: Biology, Ecology and Evolution, ed Jurkevitch E. (Berlin; Heidelberg: Springer), 213–248.

[B88] Yamaguchi T. Ikawa T. Nisizawa K. (1966). Incorporation of radioactive carbon from H^14^CO_3^−^_ into sugar constituents by a brown alga *Eisenia bicyclis* during photosynthesis and its fate in dark. Plant Cell Physiol. 7, 217–229.

[B89] Youssef N. H. Couger M. B. Elshahed M. S. (2010). Fine-scale bacterial beta diversity within a complex ecosystem (Zodletone Spring, OK, USA): the role of the rare biosphere. PLoS ONE 5:e12414. 10.1371/journal.pone.0012414 20865128 PMC2932559

[B90] Zhu M. Ghodsi A. (2006). Automatic dimensionality selection from the scree plot via the use of profile likelihood. Comput. Stat. Data Anal. 51, 918–930. 10.1016/j.csda.2005.09.010

