# Supplementary material for: Restructuring of Epibacterial Communities on Fucus vesiculosus forma mytili in Response to Elevated pCO2 and Increased Temperature Levels
Source: Front Microbiol. 2016 Mar 31;7:434. doi: 10.3389/fmicb.2016.00434 (PMC4814934; doi:10.3389/fmicb.2016.00434)
Supplement: Supplementary file 1 [file Table1.PDF]

**Tab. S1 Experimental design.** Metadata table describing the experimental design including the factors Type (2 levels: Biofilm_Fucus and Water), Week (4 levels: weeks 0, 4, 8 and 11), Temp (2 levels: 0 = ambient T and +5 = +T) and $CO_2$ (2 levels: 0 = ambient $CO_2$ and +600 = +$CO_2$).

| Sample_ID | Sample_Unit | Exp_Unit | Type | Week | Temp | $CO_2$ |
|---|---|---|---|---|---|---|
| S002 | A2_B8 | A2 | Biofilm_Fucus | 0 | +5 | +600 |
| S017 | A2_W | A2 | Water | 0 | +5 | +600 |
| S006 | C2_B8 | C2 | Biofilm_Fucus | 0 | +5 | +600 |
| S021 | C2_W | C2 | Water | 0 | +5 | +600 |
| S010 | E2_B8 | E2 | Biofilm_Fucus | 0 | +5 | +600 |
| S025 | E2_W | E2 | Water | 0 | +5 | +600 |
| S001 | A1_B8 | A1 | Biofilm_Fucus | 0 | 0 | +600 |
| S016 | A1_W | A1 | Water | 0 | 0 | +600 |
| S005 | C1_B8 | C1 | Biofilm_Fucus | 0 | 0 | +600 |
| S020 | C1_W | C1 | Water | 0 | 0 | +600 |
| S009 | E1_B8 | E1 | Biofilm_Fucus | 0 | 0 | +600 |
| S024 | E1_W | E1 | Water | 0 | 0 | +600 |
| S004 | B2_B8 | B2 | Biofilm_Fucus | 0 | +5 | 0 |
| S019 | B2_W | B2 | Water | 0 | +5 | 0 |
| S008 | D2_B8 | D2 | Biofilm_Fucus | 0 | +5 | 0 |
| S023 | D2_W | D2 | Water | 0 | +5 | 0 |
| S012 | F2_B8 | F2 | Biofilm_Fucus | 0 | +5 | 0 |
| S027 | F2_W | F2 | Water | 0 | +5 | 0 |
| S003 | B1_B8 | B1 | Biofilm_Fucus | 0 | 0 | 0 |
| S018 | B1_W | B1 | Water | 0 | 0 | 0 |
| S007 | D1_B8 | D1 | Biofilm_Fucus | 0 | 0 | 0 |
| S022 | D1_W | D1 | Water | 0 | 0 | 0 |
| S011 | F1_B8 | F1 | Biofilm_Fucus | 0 | 0 | 0 |
| S026 | F1_W | F1 | Water | 0 | 0 | 0 |
| S029 | A2_B8 | A2 | Biofilm_Fucus | 4 | +5 | +600 |
| S041 | A2_W | A2 | Water | 4 | +5 | +600 |
| S033 | C2_B8 | C2 | Biofilm_Fucus | 4 | +5 | +600 |
| S045 | C2_W | C2 | Water | 4 | +5 | +600 |
| S037 | E2_B8 | E2 | Biofilm_Fucus | 4 | +5 | +600 |
| S049 | E2_W | E2 | Water | 4 | +5 | +600 |
| S028 | A1_B8 | A1 | Biofilm_Fucus | 4 | 0 | +600 |
| S040 | A1_W | A1 | Water | 4 | 0 | +600 |
| S032 | C1_B8 | C1 | Biofilm_Fucus | 4 | 0 | +600 |
| S044 | C1_W | C1 | Water | 4 | 0 | +600 |
| S036 | E1_B8 | E1 | Biofilm_Fucus | 4 | 0 | +600 |
| S048 | E1_W | E1 | Water | 4 | 0 | +600 |
| S031 | B2_B8 | B2 | Biofilm_Fucus | 4 | +5 | 0 |
| S043 | B2_W | B2 | Water | 4 | +5 | 0 |
| S035 | D2_B8 | D2 | Biofilm_Fucus | 4 | +5 | 0 |
| S047 | D2_W | D2 | Water | 4 | +5 | 0 |
| S039 | F2_B8 | F2 | Biofilm_Fucus | 4 | +5 | 0 |
| S051 | F2_W | F2 | Water | 4 | +5 | 0 |
| S030 | B1_B8 | B1 | Biofilm_Fucus | 4 | 0 | 0 |
| S042 | B1_W | B1 | Water | 4 | 0 | 0 |
| S034 | D1_B8 | D1 | Biofilm_Fucus | 4 | 0 | 0 |
| S046 | D1_W | D1 | Water | 4 | 0 | 0 |
| S038 | F1_B8 | F1 | Biofilm_Fucus | 4 | 0 | 0 |
| S050 | F1_W | F1 | Water | 4 | 0 | 0 |

| | | | | | | |
|---|---|---|---|---|---|---|
| S065 | A2_B8 | A2 | Biofilm_Fucus | 8 | +5 | +600 |
| S077 | A2_W | A2 | Water | 8 | +5 | +600 |
| S069 | C2_B8 | C2 | Biofilm_Fucus | 8 | +5 | +600 |
| S081 | C2_W | C2 | Water | 8 | +5 | +600 |
| S073 | E2_B8 | E2 | Biofilm_Fucus | 8 | +5 | +600 |
| S085 | E2_W | E2 | Water | 8 | +5 | +600 |
| S064 | A1_B8 | A1 | Biofilm_Fucus | 8 | 0 | +600 |
| S076 | A1_W | A1 | Water | 8 | 0 | +600 |
| S068 | C1_B8 | C1 | Biofilm_Fucus | 8 | 0 | +600 |
| S080 | C1_W | C1 | Water | 8 | 0 | +600 |
| S072 | E1_B8 | E1 | Biofilm_Fucus | 8 | 0 | +600 |
| S084 | E1_W | E1 | Water | 8 | 0 | +600 |
| S067 | B2_B8 | B2 | Biofilm_Fucus | 8 | +5 | 0 |
| S079 | B2_W | B2 | Water | 8 | +5 | 0 |
| S071 | D2_B8 | D2 | Biofilm_Fucus | 8 | +5 | 0 |
| S083 | D2_W | D2 | Water | 8 | +5 | 0 |
| S075 | F2_B8 | F2 | Biofilm_Fucus | 8 | +5 | 0 |
| S087 | F2_W | F2 | Water | 8 | +5 | 0 |
| S066 | B1_B8 | B1 | Biofilm_Fucus | 8 | 0 | 0 |
| S078 | B1_W | B1 | Water | 8 | 0 | 0 |
| S070 | D1_B8 | D1 | Biofilm_Fucus | 8 | 0 | 0 |
| S082 | D1_W | D1 | Water | 8 | 0 | 0 |
| S074 | F1_B8 | F1 | Biofilm_Fucus | 8 | 0 | 0 |
| S086 | F1_W | F1 | Water | 8 | 0 | 0 |
| S101 | A2_B8 | A2 | Biofilm_Fucus | 11 | +5 | +600 |
| S116 | A2_W | A2 | Water | 11 | +5 | +600 |
| S105 | C2_B8 | C2 | Biofilm_Fucus | 11 | +5 | +600 |
| S120 | C2_W | C2 | Water | 11 | +5 | +600 |
| S109 | E2_B8 | E2 | Biofilm_Fucus | 11 | +5 | +600 |
| S124 | E2_W | E2 | Water | 11 | +5 | +600 |
| S100 | A1_B8 | A1 | Biofilm_Fucus | 11 | 0 | +600 |
| S115 | A1_W | A1 | Water | 11 | 0 | +600 |
| S104 | C1_B8 | C1 | Biofilm_Fucus | 11 | 0 | +600 |
| S119 | C1_W | C1 | Water | 11 | 0 | +600 |
| S108 | E1_B8 | E1 | Biofilm_Fucus | 11 | 0 | +600 |
| S123 | E1_W | E1 | Water | 11 | 0 | +600 |
| S103 | B2_B8 | B2 | Biofilm_Fucus | 11 | +5 | 0 |
| S118 | B2_W | B2 | Water | 11 | +5 | 0 |
| S107 | D2_B8 | D2 | Biofilm_Fucus | 11 | +5 | 0 |
| S122 | D2_W | D2 | Water | 11 | +5 | 0 |
| S111 | F2_B8 | F2 | Biofilm_Fucus | 11 | +5 | 0 |
| S126 | F2_W | F2 | Water | 11 | +5 | 0 |
| S102 | B1_B8 | B1 | Biofilm_Fucus | 11 | 0 | 0 |
| S117 | B1_W | B1 | Water | 11 | 0 | 0 |
| S106 | D1_B8 | D1 | Biofilm_Fucus | 11 | 0 | 0 |
| S121 | D1_W | D1 | Water | 11 | 0 | 0 |
| S110 | F1_B8 | F1 | Biofilm_Fucus | 11 | 0 | 0 |
| S125 | F1_W | F1 | Water | 11 | 0 | 0 |
