# Supplementary material for: Restructuring of Epibacterial Communities on Fucus vesiculosus forma mytili in Response to Elevated pCO2 and Increased Temperature Levels
Source: Front Microbiol. 2016 Mar 31;7:434. doi: 10.3389/fmicb.2016.00434 (PMC4814934; doi:10.3389/fmicb.2016.00434)
Supplement: Supplementary file 2 [file Table2.PDF]

Tab. S2 Level-wise evaluation. Results of the level-wise evaluation of significant interaction terms ("Type:Temp:Week" and "CO2:Week") based on Redundancy Analysis (RDA). F-, *p*- and *q*-values (Benjamini-Hochberg-adjusted *p*-values) as well as adjusted R^2 values are given for each evaluated term, along with the RDA model formula applied for testing. $F_{(dfn,dfd)}$, F value with numerator and denominator degrees of freedom; TOC, transformed OTU counts; Temp, Temperature.

Evaluated Term	RDA model formula	$F_{(dfn,dfd)}$	*p*	*q*	adjusted R^2
**Temp:Week within constant Type**	**TOC ~ Temp:Week + Condition(Temp + CO_2 + Week + CO_2:Week)**	$F_{3,36}$			
Biofilm_Fucus.Temp:Week		1.66	0.006	0.012	0.03
Water.Temp:Week		1.66	0.015	0.015	0.02
**Temp within constant Type and Week**	**TOC ~ Temp + Condition(CO_2)**	$F_{1,9}$			
Biofilm_Fucus.Temp.Week_00		0.96	0.509	0.509	0.00
Biofilm_Fucus.Temp.Week_04		2.15	0.007	0.009	0.10
Biofilm_Fucus.Temp.Week_08		1.75	0.002	0.004	0.07
Biofilm_Fucus.Temp.Week_11		2.04	0.001	0.004	0.10
Water.Temp.Week_00		0.94	0.549	0.549	-0.01
Water.Temp.Week_04		3.16	0.004	0.016	0.18
Water.Temp.Week_08		2.02	0.008	0.016	0.09
Water.Temp.Week_11		1.53	0.074	0.099	0.05
**CO_2 within constant Week**	**TOC ~ CO_2 + Condition(Type + Temp + Type:Temp)**	$F_{1,19}$			
CO_2.Week_00		1.04	0.406	0.406	0.00
CO_2.Week_04		1.13	0.266	0.355	0.00
CO_2.Week_08		1.44	0.014	0.056	0.01
CO_2.Week_11		1.31	0.079	0.158	0.01
