# Supplementary material for: Restructuring of Epibacterial Communities on Fucus vesiculosus forma mytili in Response to Elevated pCO2 and Increased Temperature Levels
Source: Front Microbiol. 2016 Mar 31;7:434. doi: 10.3389/fmicb.2016.00434 (PMC4814934; doi:10.3389/fmicb.2016.00434)
Supplement: Supplementary file 3 [file Table3.PDF]

Tab. S3 Taxonomy of OTUs in OTU association network (see **Fig. S6**).

OTU	Phylum	Class	Order	Family	Genus
1	Proteobacteria	Alphaproteobacteria	Rickettsiales	Pelagibacteraceae	uncl. Pelagibacteraceae
2	Bacteroidetes	Flavobacteriia	Flavobacteriales	Flavobacteriaceae	Sediminicola
3	Proteobacteria	Alphaproteobacteria	Rhodobacterales	Rhodobacteraceae	Octadecabacter
4	Bacteroidetes	Flavobacteriia	Flavobacteriales	Cryomorphaceae	uncl. Cryomorphaceae
5	Proteobacteria	Gammaproteobacteria	Alteromonadales	Alteromonadaceae	Glaciecola
6	Proteobacteria	Gammaproteobacteria	Oceanospirillales	Halomonadaceae	Candidatus Portiera
7	Proteobacteria	Gammaproteobacteria	uncl. Gammaproteobacteria	uncl. Gammaproteobacteria	uncl. Gammaproteobacteria
9	Proteobacteria	Alphaproteobacteria	Rhodobacterales	Rhodobacteraceae	Octadecabacter
10	Proteobacteria	Gammaproteobacteria	Alteromonadales	HTCC2188	HTCC
15	Proteobacteria	Alphaproteobacteria	Rhodobacterales	Rhodobacteraceae	uncl. Rhodobacteraceae
16	Proteobacteria	Gammaproteobacteria	Alteromonadales	Alteromonadaceae	Glaciecola
21	Proteobacteria	Alphaproteobacteria	Rhodobacterales	Rhodobacteraceae	uncl. Rhodobacteraceae
22	Bacteroidetes	Saprospirae	Saprospirales	Saprospiraceae	uncl. Saprospiraceae
24	Proteobacteria	Alphaproteobacteria	Rhodobacterales	Rhodobacteraceae	uncl. Rhodobacteraceae
26	Proteobacteria	Alphaproteobacteria	Rhodobacterales	Rhodobacteraceae	uncl. Rhodobacteraceae
27	Proteobacteria	Gammaproteobacteria	Oceanospirillales	Halomonadaceae	Halomonas
45	Bacteroidetes	BME43	uncl. BME43	uncl. BME43	uncl. BME43
58	Bacteroidetes	Flavobacteriia	Flavobacteriales	Flavobacteriaceae	Polaribacter
88	Proteobacteria	Alphaproteobacteria	Rhodobacterales	Rhodobacteraceae	uncl. Rhodobacteraceae
90	Proteobacteria	Alphaproteobacteria	Sphingomonadales	uncl. Sphingomonadales	uncl. Sphingomonadales
92	Proteobacteria	Betaproteobacteria	Burkholderiales	Comamonadaceae	uncl. Comamonadaceae
100	Proteobacteria	Alphaproteobacteria	Rhodobacterales	Rhodobacteraceae	uncl. Rhodobacteraceae
165	Proteobacteria	Alphaproteobacteria	uncl. Alphaproteobacteria	uncl. Alphaproteobacteria	uncl. Alphaproteobacteria
177	Proteobacteria	Betaproteobacteria	Burkholderiales	Oxalobacteraceae	Herbaspirillum
208	Proteobacteria	Alphaproteobacteria	Rhizobiales	Bradyrhizobiaceae	Afipia
