# Supplementary material for: Restructuring of Epibacterial Communities on Fucus vesiculosus forma mytili in Response to Elevated pCO2 and Increased Temperature Levels
Source: Front Microbiol. 2016 Mar 31;7:434. doi: 10.3389/fmicb.2016.00434 (PMC4814934; doi:10.3389/fmicb.2016.00434)
Supplement: Supplementary file 4 [file Image1.PDF]

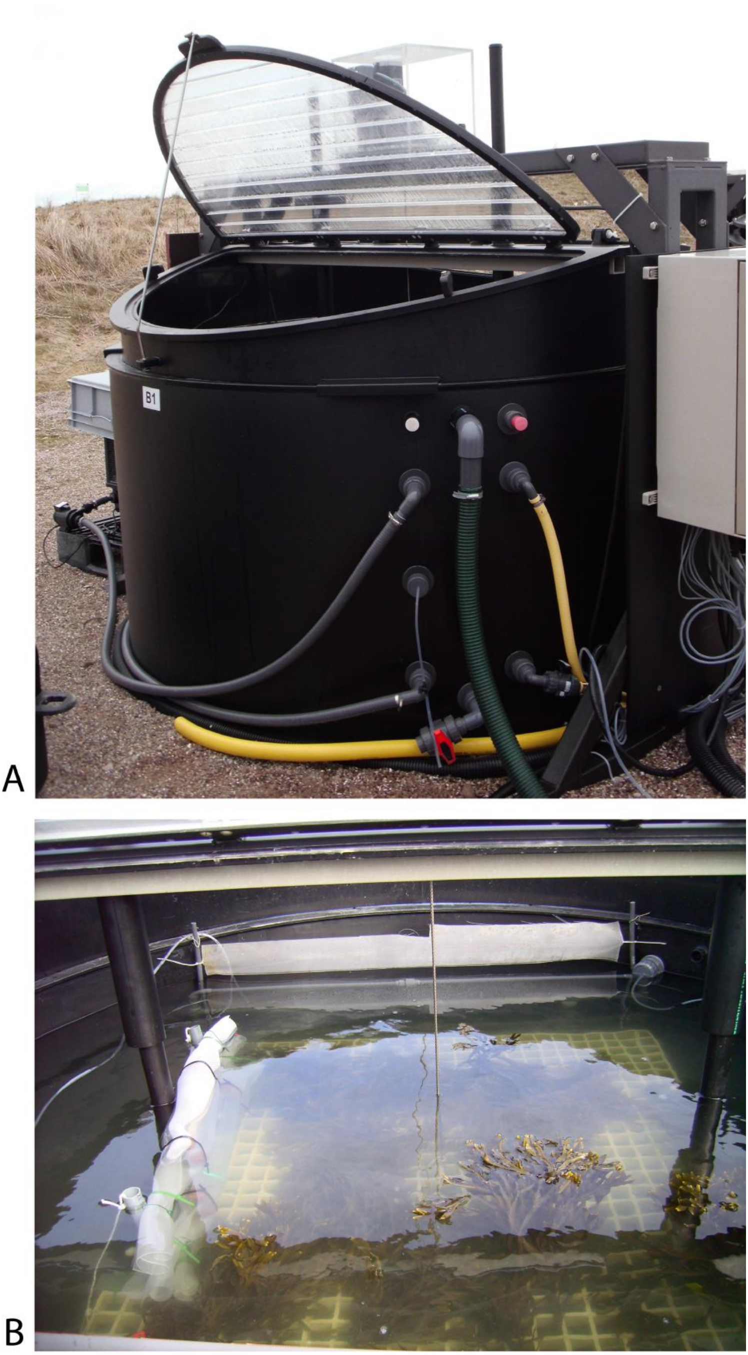

Fig. S1 Benthic mesocosm facility on the island of Sylt (List, Germany). (A) One of the 12 mesocosms with opened lid. (B) View inside one mesocosms onto the vertically adjustable grating populated with 11 individuals of *Fucus mytili*.
