# Supplementary material for: Restructuring of Epibacterial Communities on Fucus vesiculosus forma mytili in Response to Elevated pCO2 and Increased Temperature Levels
Source: Front Microbiol. 2016 Mar 31;7:434. doi: 10.3389/fmicb.2016.00434 (PMC4814934; doi:10.3389/fmicb.2016.00434)
Supplement: Supplementary file 5 [file Image2.PDF]

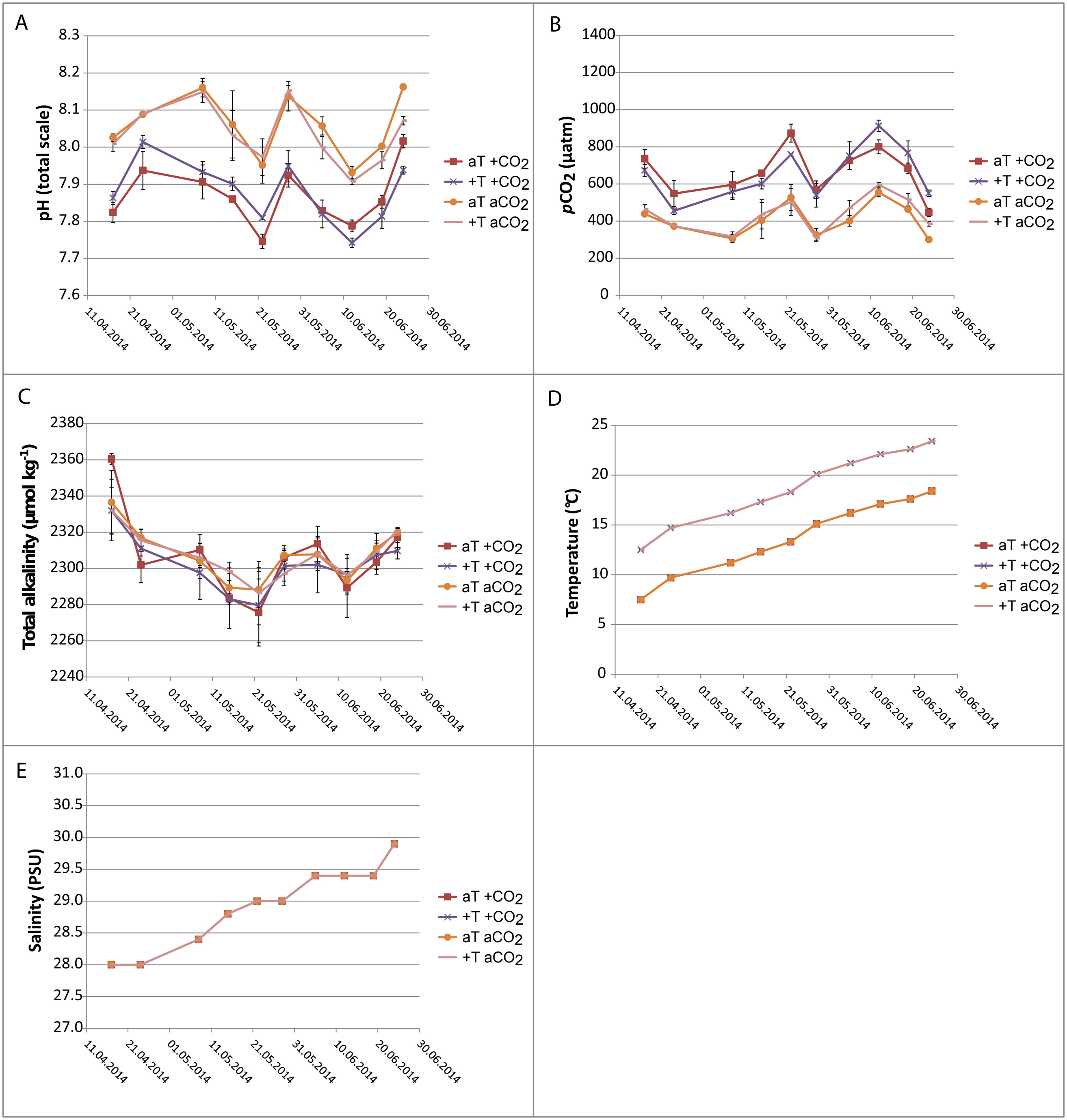

Fig. S2 Seawater parameters of the benthic mesocosm experiment. (A) pH at total scale, (B) calculated pCO_2 in µatm, (C) total alkalinity in µmol kg^{-1}, (D) simulated seawater temperature in °C, and (E) salinity in PSU, measured at a weekly basis, respectively. For different temperature and pCO_2 conditions see 'Materials and Methods' section; a, ambient; +, increased/elevated.
