# Supplementary material for: Restructuring of Epibacterial Communities on Fucus vesiculosus forma mytili in Response to Elevated pCO2 and Increased Temperature Levels
Source: Front Microbiol. 2016 Mar 31;7:434. doi: 10.3389/fmicb.2016.00434 (PMC4814934; doi:10.3389/fmicb.2016.00434)
Supplement: Supplementary file 6 [file Image3.PDF]

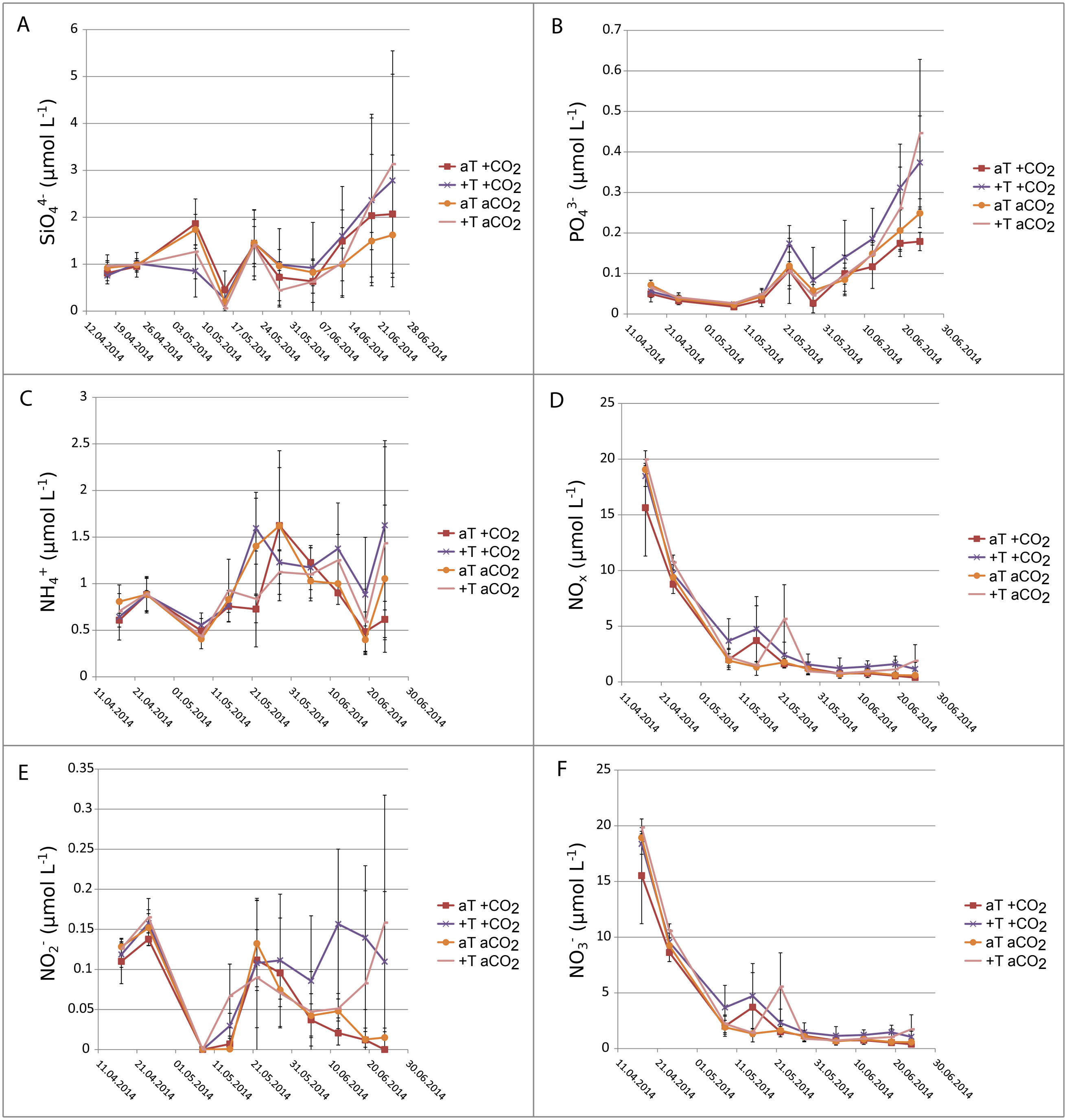

Fig. S3 Seawater inorganic nutrients from the benthic mesocosm experiment. (A) silicate (SiO_4^{4-}), (B) phosphate (PO_4^{3-}), (C) ammonium (NH_4^+), (D) total nitrogen oxide (NO_x), (E) nitrite (NO_2^-), and (F) nitrate (NO_3^-, calculated as $NO_x - NO_2^-$) concentrations in µmol L^{-1} and measured at a weekly basis, respectively. For different temperature and $p$$CO_2$conditions see 'Materials and Methods' section; a, ambient; +, increased/elevated.
