# Supplementary material for: Restructuring of Epibacterial Communities on Fucus vesiculosus forma mytili in Response to Elevated pCO2 and Increased Temperature Levels
Source: Front Microbiol. 2016 Mar 31;7:434. doi: 10.3389/fmicb.2016.00434 (PMC4814934; doi:10.3389/fmicb.2016.00434)
Supplement: Supplementary file 7 [file Image4.PDF]

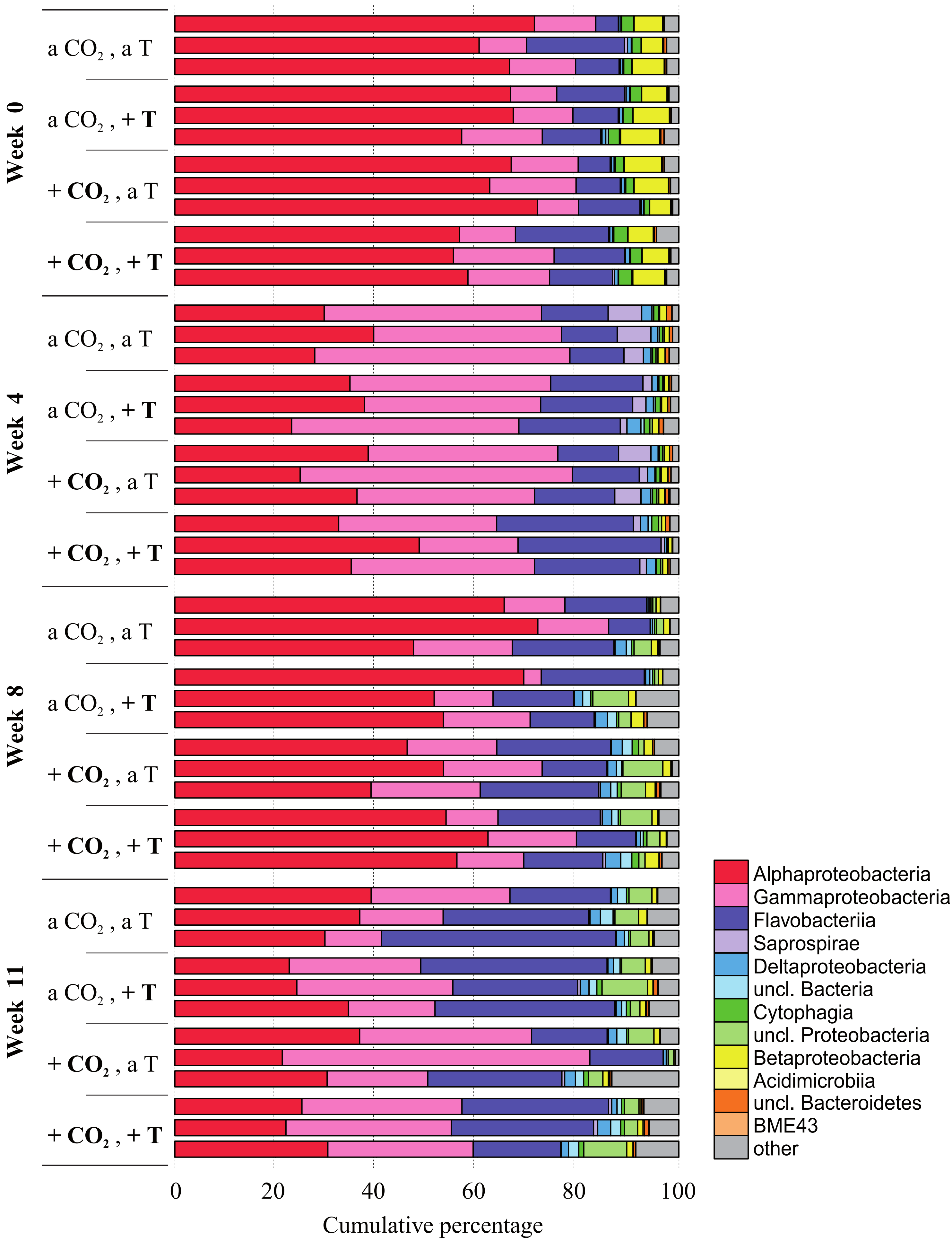

Fig. S4 Relative abundances (in cumulative percentage) of the most abundant bacterial classes in seawater. Bacterial community compositions were shown for each sample (n=3 per treatment) after 0, 4, 8 and 11 weeks of incubation in the benthic mesocosms under different conditions (see 'Materials and Methods' section; a, ambient; +, elevated). Analysis is based on bacterial 16S rDNA (V1-V2) amplicon sequences (Illumina MiSeq). Low abundant classes (≤1%) were summarized as "other".
