# Supplementary material for: Restructuring of Epibacterial Communities on Fucus vesiculosus forma mytili in Response to Elevated pCO2 and Increased Temperature Levels
Source: Front Microbiol. 2016 Mar 31;7:434. doi: 10.3389/fmicb.2016.00434 (PMC4814934; doi:10.3389/fmicb.2016.00434)
Supplement: Supplementary file 8 [file Image5.PDF]

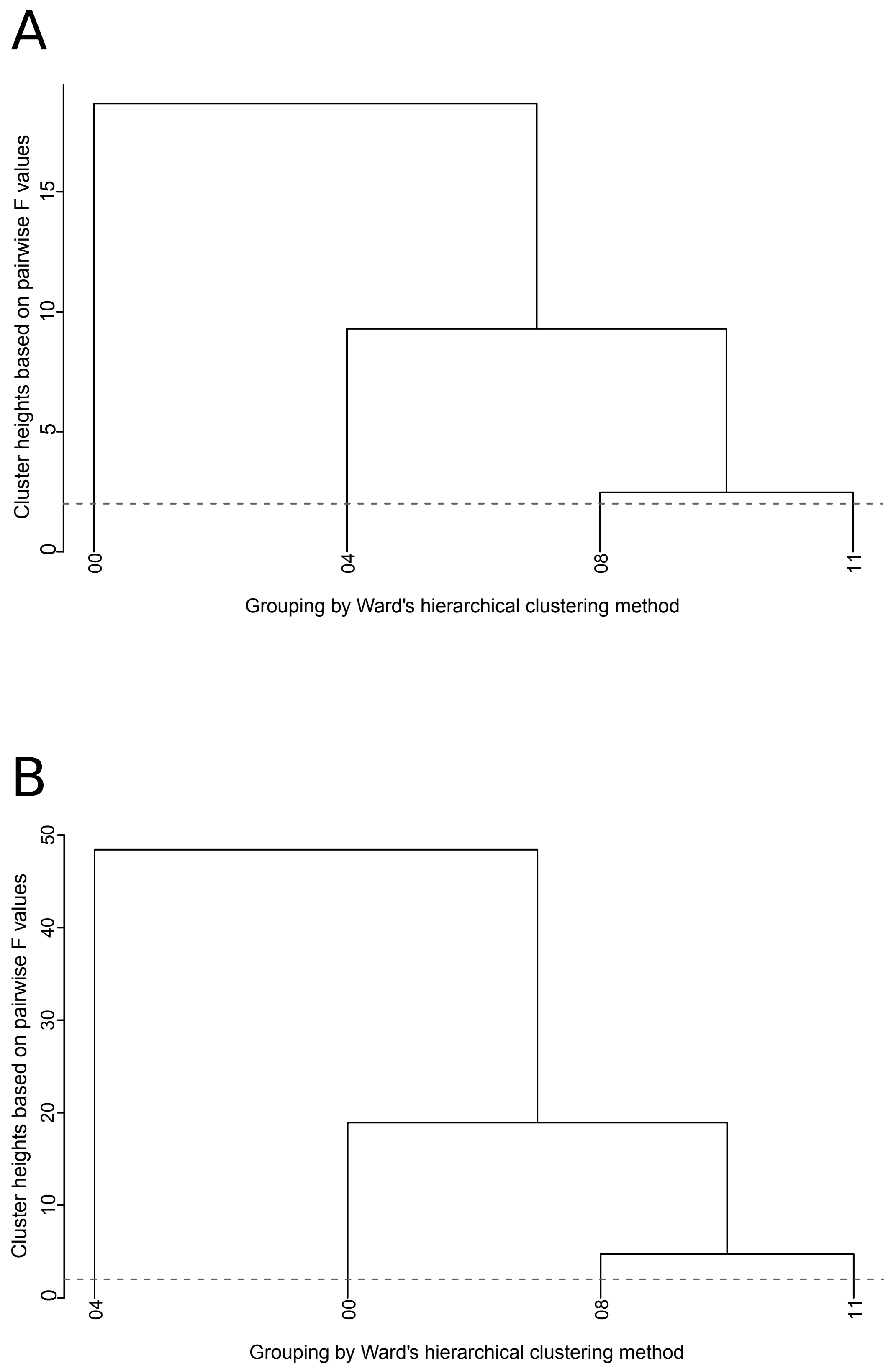

Fig. S5 Week-wise evaluation of community patterns. Clustering of Week levels for (A) *Fucus mytili* biofilm and (B) water samples: Grouping by Ward's hierarchical clustering method (for Weeks 00, 04, 08, 11) with Cluster heights based on pairwise F values. F≥2 indicates a threshold (dotted line) with bacterial communities differing markedly.
