# Supplementary material for: Restructuring of Epibacterial Communities on Fucus vesiculosus forma mytili in Response to Elevated pCO2 and Increased Temperature Levels
Source: Front Microbiol. 2016 Mar 31;7:434. doi: 10.3389/fmicb.2016.00434 (PMC4814934; doi:10.3389/fmicb.2016.00434)
Supplement: Supplementary file 9 [file Image6.PDF]

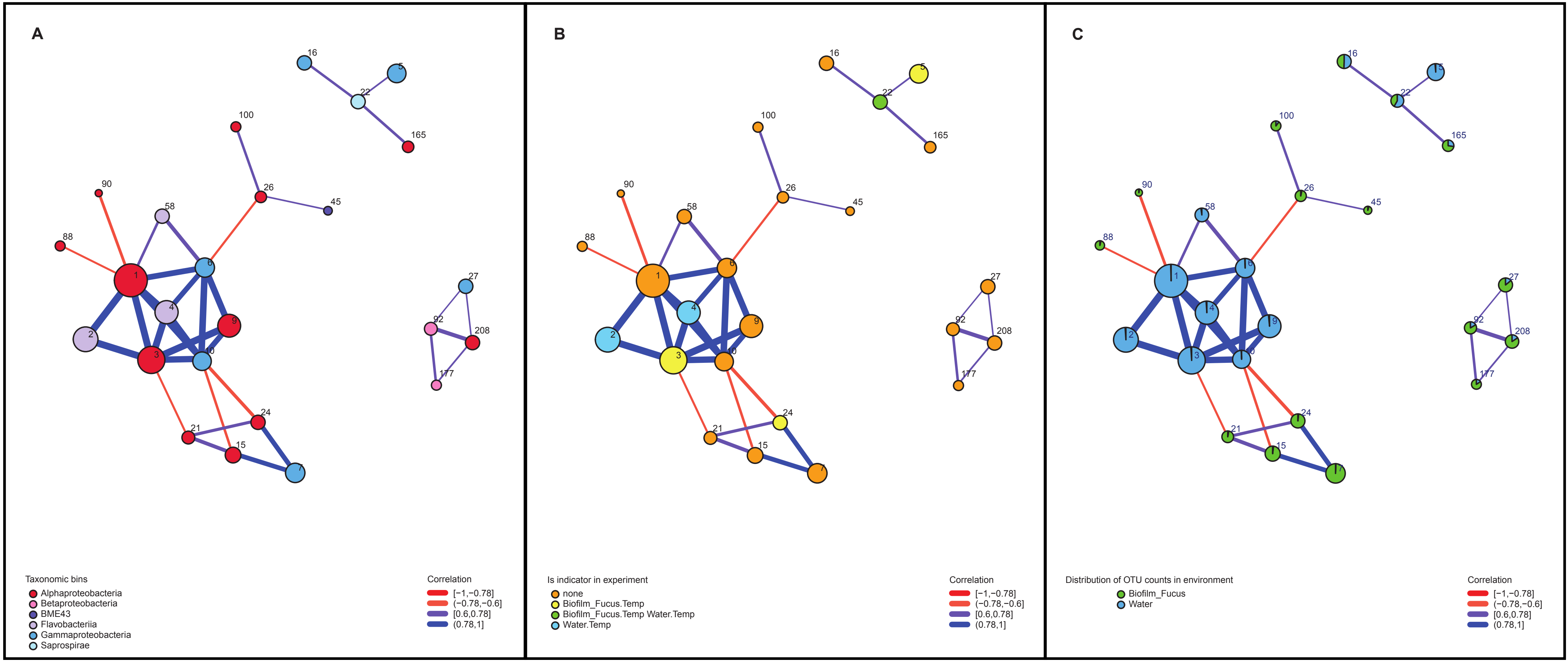

Fig. S6 Association network of strongly correlated OTUs. Vertices represent OTUs and are labeled by OTU number (see **Tab. S3**) and colored according to (A) class-level taxonomy, (B) indicator property, or (C) environmental distribution, respectively. Width and color of the edges connecting the vertices vary according to strength and sign of the correlation between associated OTUs. Diameters of the vertices vary according to the relative abundance of each OTU.
